# Extensions to Michaelis-Menten Kinetics for Single Parameters

**DOI:** 10.1038/s41598-018-34675-2

**Published:** 2018-11-08

**Authors:** R. T. K. Ariyawansha, B. F. A. Basnayake, A. K. Karunarathna, M. I. M. Mowjood

**Affiliations:** 1 Postgraduate Institute of Agriculture, University of Peradeniya, Peradeniya, 20400 Sri Lanka; 2 Department of Agricultural Engineering, Faculty of Agriculture, University of Peradeniya, Peradeniya, 20400 Sri Lanka

## Abstract

Biochemical transformation kinetics is based on the formation of enzyme-substrate complexes. We developed a robust scheme based on unit productions of enzymes and reactants in cyclic events to comply with mass action law to form enzyme-substrate complexes. The developed formalism supports a successful application of Michaelis-Menten kinetics in all biochemical transformations of single parameters. It is an essential tool to overcome some challenging healthcare and environmental issues. In developing the formalism, we defined the substrate [S]= [*Product*]^*3/4*^ and rate of reaction based on rate and time perspectives. It allowed us to develop two quadratic equations. The first, represents a body entity that gave a useful relationship of enzyme *E* = 2*S*^0.33^, and the second nutrients/feed, each giving [*Enzymes*] and [*Enzyme-substrate complexes*], simulating rate of reaction, [*substrate*], and their differentials. By combining [*Enzymes*] and [*Enzyme-substrate complexes*] values, this quadratic equation derives a Michaelis-Menten hyperbolic function. Interestingly, we can derive the proportionate rate of reaction and [*Enzymes*] values of the quadratics resulting in another Michaelis-Menten hyperbolic. What is clear from these results is that between these two hyperbolic functions, in-competitive inhibitions exist, indicating metabolic activities and growth in terms of energy levels. We validated these biochemical transformations with examples applicable to day to day life.

## Introduction

Modern lifestyles resulting from rapid development may have detrimental health and environmental impacts, often resulting in non-communicable diseases, and may also adversely affect the equilibrium of the biosphere of planet earth. Biochemical transformation (BT) kinetics is a ubiquitous parameter in biospheres and features in multiple interactions among its biotic components of plants, animals, and micro-organisms^1,2^. Thus far, several studies have attempted to understand and define BT kinetics of the biosphere by developing mathematical kinetics models^3–9^. The seminal Michaelis-Menten (MM) scheme, although a century old, is the basic enzyme kinetics model which has been extensively used in biochemistry for studying enzymatic catalysis^3,9–12^. Of importance is the formation of enzyme-substrate complex (*ES*) in MM kinetics that is fundamental to our understanding of the MM scheme and it was validated by Chance^11^. One study in particular combined MM kinetics with growth of the mammals^13^, and in parallel, several mathematical models were developed based on single parameters to determine the growth of mammals, plants, and microbial activity^13–16^. From all the above studies, we found the model developed by West and co-workers to be unique^15^. Their distinctive findings combined with MM kinetics are the source of our interest and it has resulted in us developing a tool for analysis. It is possible to hypothetically obtain values of enzymes (*E*) and enzyme-substrate complexes from our generalized formulism applicable to all types of BTs.

In our study, we were motivated by the numerous interpretations of the original works of MM, but because MM’s findings have not been extensively examined, even for analysing single parameters. We believe an extension to MM kinetics will provide another approach to analysing any BTs. So far, many of the researchers have relied on Briggs-Haldane’s kinetics module, which is widely adopted to delineate a reduction in the substrate (*S*) while the product (*P*) increases^10^. Briggs-Haldane’s kinetics module is stated as;

1 $${\rm{E}}+{\rm{S}}\underset{{k^{\prime} }_{1}}{\overset{{k}_{1}}{\rightleftharpoons }}{\rm{ES}}\,\mathop{\longrightarrow }\limits^{{k}_{2}}\,{\rm{P}}+{\rm{E}}$$

2 $$\frac{d[S]}{dt}=-\,{k}_{1}[E][S]+{k}_{1}^{^{\prime} }[ES]$$

3 $$\frac{d[E]}{dt}=-\,{k}_{1}[E][S]+{k}_{1}^{^{\prime} }[ES]+{k}_{2}[ES]$$

4 $$\frac{d[ES]}{dt}={k}_{1}[E][S]-{k}_{1}^{^{\prime} }[ES]-{k}_{2}[ES]$$

5 $$\frac{d[P]}{dt}={k}_{2}[ES]$$

where, *t* = time and rate constants of *k*_1_, ${k^{\prime} }_{1}$, and *k*_2_

In this scheme, equations (2) and (4) becomes similar but with an opposite direction of the overall reaction rate *v*, when the equation (4) reaches a steady state where, $\frac{d[ES]}{dt}=0$. Interestingly, $\frac{d[P]}{dt}=v=-\,\frac{d[S]}{dt}$, most authors give a positive value for the change in the substrate rate^6,17–20^. Regardless of such inequalities, many researchers have denoted $v=\frac{d[S]}{dt}$ or $v=\frac{d[P]}{dt}$ and substrate *S*^17^ while consuming and then obtaining *v*_*m*_ and *K*_*m*_ values using Lineweaver-Burke to plot $\frac{1}{v}\,vs\,\,\frac{1}{[S]}$ ^18,21–23^ or Eddie Hofstee plot it as $v\,\,vs\frac{v}{[S]}$ ^23^. Whenever, experimental values differed from obtaining a hyperbolic function, additional constants of *k* were included, apart from *K*_*m*_ to substantiate the variations from MM kinetics^24,25^. Some developed mathematical models both at micro^24–29^ and macroscopic levels^21,24^ deviating from MM, and some included appendages to MM^18,25,30–33^ as a means of providing a scientific explanation to some of the research findings.

**Figure 1:**
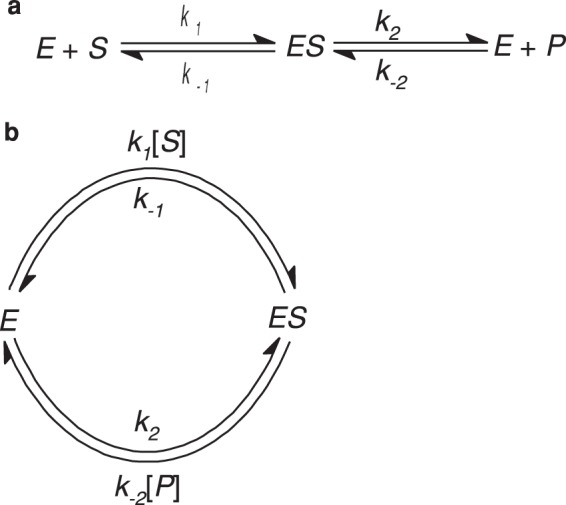
The simple, classic enzyme kinetic scheme (**a**) corresponds to a cyclic reaction of a single enzyme in (**b**)^28^.

We understand from a thermodynamic and biological point of view, Briggs-Haldane’s is an open system, while MM kinetics is a closed system that increases both substrate and overall reaction rate *v* to follow a hyperbolic function when they are^18,23,34^. We found that such behaviour can only be expected, if the substrate is in excess without contravening mass action law, thus clearly redefining $\frac{d[S]}{dt}$. Such a condition can only be expected from BT cyclic paths well recognized by many studies^25,27,28,31,35–37^ (Fig. 1) which provides a sound solution in the use of MM kinetics. One of the prerequisites within MM is the aspect of “time dependency” in *v*^28^, which commence from the start of the first cycle, influencing overall rate of reaction, thus enabling determination of enzyme-substrate complexes. Such a study was carried out by Kosmidis and co-workers^25^, where they modified MM equation by incorporating the “time dependence” in the Michaelian “constant”, at the macroscopic level.

We understand that one of the limitations when using MM kinetics is the accuracy needed to determine with some precision the actual quantities of substrate and enzymes consumed in reactions. It is reported^38^ that there are complexities in isolating enzymes and in reality, BTs occur not in its purest form. To overcome the above, we analysed the product, which is far more convenient, absolute, and accessible from massive databases that exist currently in different fields of study. We propose a formalism which allows a comparison of many of the inputs like plant nutrients, changes in diets of humans and animals, dispensation of medication, reduction of pollution with an ability to monitor hypothetical enzymes, enzyme-substrate complexes, energy levels, and all other inner mechanisms with far fewer interventions, thereby enabling improvements to biosystems, ecosystems, through the development of new reactors, while at the same time providing some solutions to health issues.

## Extension to MM Kinetics

The challenge we faced in our attempts to use MM kinetics were to identify the terms *v* and *S*, the substrate itself for enzymatic reactions. In other words, when we are unable to know the actual substrate and the enzyme as the parameters, particularly for a single parameter like the growth of a plant, we do need to make some assumptions, like, [*P*] = mass, plant height or concentration and that [*S*] = [*P*]^*n*^. Critically, *n* is not known. While this fact remained an unresolved problem, we chose to examine closely the research on *M*^3/4^ relationship, where *M* = mass of the mammal in which the hypothesis has been supported by researchers^14,15,39–43^. The general model developed by West and co-workers for ontogenetic growth based on the conservation of energy equation can be applied to biomass generations^15^, thus [*P*] = *M*. Therefore, we can assume that [*P*]^3/4^ = [*S*]. In applying to microbial populations, it has been reported that the active mass is approximately 20% (w/w) of volatile suspended solids (VSS)^1^. Therefore, when we evaluated our data of different studies influenced by microbial populations, we can obtain the availability of [*S*] by applying the rule *M*^3/4^, because $\frac{{M}^{3/4}}{M}=\frac{[S]}{[P]}$ in most cases approaches the value 0.2 or ≥0.2 of VSS or volatile solids (VS) (Table S1).

**Figure 2:**
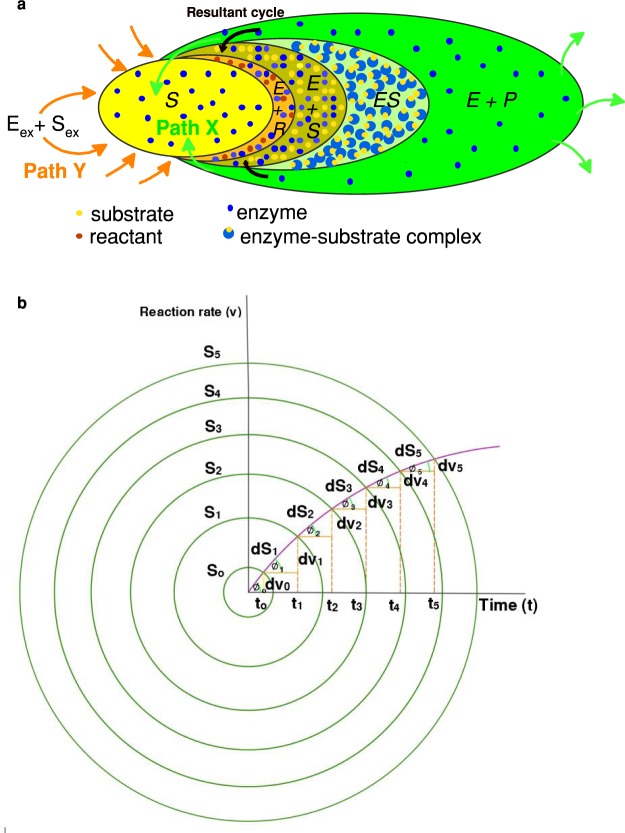
The relationship between variables. (**a**) The illustration of the cyclic nature of forming enzymes from substrates, enzyme-substrate complexes, and products. This also shows thermodynamically heterogeneous reactions while kinetically homogenous behaving in a close loop cycle. It coincides with steps in equation (6), where *S* = substrate (A), *E* + *R* = substrate + reactants (B), *E* + *S* = enzymes + substrates (C), *ES* = enzyme-substrate complexes (D), and *E* + *P* = enzymes + products (E) (**b**) Defining of *v*. The relationships between the variables *dv*, *d*[*S*], and *t* in equation (21). In which the progressive increment of *v* with time is enveloped with the heterogeneous substrate increases of *d*[*S*]. The substrate concentration increases from *S*_1_ *to S*_2_ for the time duration from *t*_1_ to *t*_2_, corresponding to intersects of *v*_1_ and *v*_2_.

In defining $\frac{d[S]}{dt}$, it is a diminishing term as expressed by Briggs-Haldane. This is also the case with mass conservation law, $\frac{d[S]}{dt}\ne \frac{d[P]}{dt}$. Therefore, there should be adequate supply of *E* + *S* to bring about an equilibrium state, thus making $\frac{d[S]}{dt}=0$. However, like $\frac{d[P]}{dt}$, $\frac{d[S]}{dt}$ cannot be zero during biochemical transformations. Inevitably, the forward reaction *k*_1_ should be $\frac{d[S]}{dt}$. Thus, $\frac{d[S]}{dt}={k}_{1}$. An extension to MM is conceptually perceived to accommodate zero order expression as A to B in equation (6). The cyclic nature of enzyme-substrate complexes and products can be used as the basis for developing the proposed scheme. We employ the concept of a unit production to describe the cyclic behaviour of the reactions as stated in equation (6) (Fig. 2(a)).6where, *R* = reactant (Supplementary Text 1), *k*_1_, ${k^{\prime} }_{1}$, and *k*_2_ = rate constants, path *X* = substrates from internal body entity, path *Y* = substrates from food and nutrients supply.

The unit production is considered at the start of each cycle from *A* to *B*.

7 $$\frac{d[S]}{dt}=-\,{k}_{1}[S]+{k}_{1}[E][R]$$

8 $$[E][R]-[S]=1$$

The type and form of substrate govern the path of the reactions, since enzymes are derived from both products and substrates^37,44–46^ at the start of each cycle. Therefore, it is the rate of reaction of substrates that will govern the rate of enzyme secretions, while in equilibrium, because the steps from *A* to *B* to *C* are reactions in series of the cycles with defined unit production, leading to;

9 $$\frac{d[R]}{dt}=-\,{k}_{1}[E][R]+{k}_{r}[E][S]+{k}_{1}[S]$$

At equilibrium, $\frac{d[R]}{dt}=0$ and considering equation (8),

10 $${k}_{r}=\frac{{k}_{1}}{[E][S]}$$

11 $${K}_{S}=\frac{{k}_{1}}{{k}_{r}}=[E][S]$$

Notably *K*_*S*_, the dissociation constant manifests very high values compared to *k*_1_, because a unit production is involved in each cycle, thus ensuring production of equal amounts of *E* and *S*. Similar to the reaction between *B* and *C*, the forward rate of reaction between *C* and *D* should be *k*_1_ since, *k*_*r*_ < *k*_1_ and *k*_*r*_ < 1. Also, dissociation coefficient of *K*_*S*_ and *K*_*m*_ should be much larger than *k*_1_.

Henceforth, the classical MM kinetics is applied to the considered cycle from *C* to *D* to *E*.

At this equilibrium, steady state conditions will prevail, where $\frac{d[ES]}{dt}=0$,

12 $${k}_{1}[E][S]=({k}_{1}^{^{\prime} }+{k}_{2})[ES]$$

By calling the initial concentration of enzyme,

13 $$[{E}_{o}]=[ES]+[E]$$

14 $$v={k}_{2}[ES]$$

15 $${v}_{m}={k}_{2}[{E}_{o}]$$

16 $${\rm{And}}\,{K}_{m}=\frac{{k}_{1}^{^{\prime} }+{k}_{2}}{{k}_{1}}$$

17 $${\rm{Also}},\,{K}_{m}=\frac{[E][S]}{[ES]}$$

18 $${\rm{Thus}},\,v=\frac{{v}_{m}[S]}{{K}_{m}+[S]}$$

where; *v*_*m*_ = maximum rate of reaction, *K*_*m*_ = MM constant^3,18^.

The relationship between *k*_1_ and *k*_2_ is found by considering the resultant (equivalent) cyclic effect from *D* to *E* to *C* to *D*. This resultant is an outcome of the actual path *D* to *E* to *A* to *B* to *C* to *D*, which creates an equilibrium between the products and substrates. It takes into account external supply of substrates and enzymes which are imbedded in *S*. In effect, *ES* complexes create unidirectional movement at the rate of *k*_2_ to form products and enzymes. Therefore, the resultant generation rate of *E* + *S* from *E* + *P* should then be *k*_2_. *k*_1_ could be considered as the reversible resultant, having a counter movement to *k*_2_, since *k*_1_ is the driving force governing a unit production and establishing an equilibrium state, thus making $\frac{d[R]}{dt}=0$ in equation (9) for supplying paired enzymes and substrates to forming enzyme-substrate complexes. The mass action law applied to the resultant cycle *D* to *E* to *C* to *D* can be written as;

19 $$\frac{d[P]}{dt}=-\,{k}_{2}[P][E]+{k}_{2}[ES]+{k}_{1}[E][S]$$

At equilibrium, $$\frac{d[P]}{dt}=0$$.

It leads to the solution (Supplementary Method 2)

20 $${k}_{2}=\frac{{k}_{1}[S]}{[P]}$$

20a $${\rm{Also}},\,{K}_{e}=\frac{{k}_{1}}{{k}_{2}}={S}^{0.333}$$

We will see later that *K*_*e*_ the dissociation constant is directly related to enzymes. In this enzyme cycle, forward rate reactions are *k*_1_ and *k*_2_, and product formations are governed by the overall rate of reaction *v*. Therefore, it is the controlling rate of reaction from commencement of reactions at the start of first cycle to the end of reactions, thus going through all of the cycles, involving ‘time perspective’. Such a phenomenon has gone unnoticed from the findings of MM, because most researchers, like Hong Qian^28^ viewed MM kinetics to be based on ‘rate perspective’. But interestingly, Hong Qian did find that the ‘time perspective’ was equivalent in principle to that of MM in Qian’s study of single-molecule enzyme cyclic kinetics based on the theory originated by Van Slyke and Cullen in 1914.

In defining the term *v*, the initial conditions are defined as zero before any enzyme catalytic reactions can occur^17,47^. Thus, both the enzymes and substrates have to have the same time origin but progressively undergo reactions at defined rates. In other words, rate perspective of $\frac{dv}{dt}$ and $\frac{d[S]}{dt}$ should be combined with time, considering the time perspectives. Thus,

21 $$\frac{dv}{dt}=\frac{d[S]}{dt}\times \frac{1}{t}\,{\rm{and}}\,\frac{dv}{d[S]}=\frac{1}{t}$$

The relationships between the variables *dv*, *d*[*S*], and *t* in equation (21) are illustrated in Fig. 2(b). Where *v* can be written as;

22 $$v=\int dv=\int \frac{d[S]}{dt.t}\,.dt$$

It is a very difficult function to resolve. However, the analysis of the change in angle *ϕ* in Fig. 2(b) could be a means by which to explain the influence of time dependency. This angle is the reciprocal value stated in the original paper of MM as the angle of rotation. The analysis of the data obtained by MM shows a direct proportionality between angle of rotation and absolute time, particularly so at higher concentrations of substrates. One of the analysed results of MM approaches the unique value found in our study of $\frac{1}{\varphi }=0.017t$ applicable to all cases. It implies that immaterial of the differential values of $\frac{d[S]}{dt}$ and $\frac{dv}{dt}$, the rotational angle $(\frac{1}{\varphi })$ is proportional to time, on condition that the supply of enzymes and substrates is in equilibrium with the production of enzyme-substrate complexes. Such conditions will always exist in BTs, even at lower concentrations, because of the cyclic nature of the reactions in supplying adequate substrates and enzymes. It is unlike the actual experimental procedure required to determine *v* at different concentrations. Therefore, an approximate integral can be derived by considering the change in angle of *ϕ* to deduce *v*. It follows that equation (21) can be also be written as;

23 $$dv=d[S]\sin (\varphi )$$

If equation (23) is divided by *dϕ*

24 $$\frac{dv}{d\varphi }=\frac{d[S]}{d\varphi }\,.\,\sin (\varphi )\,{\rm{and}}\,{\int }_{0}^{v}dv={\int }_{{\varphi }_{1}}^{{\varphi }_{2}}\frac{d[S]}{({\varphi }_{1}-{\varphi }_{2})}\,.\,\sin \,\varphi .d\varphi $$

25 $$\mathrm{Therefore},\,v=-\,d[S]\,.\,\mathrm{ln}({\varphi }_{1}-{\varphi }_{2})\,.\,\cos (\varphi )+C$$

Note that *ϕ*_1_ > *ϕ*_2_ and also cos(*ϕ*) ≈ 1 for very small angles.

Since the integral of *d*[*S*] is not known, the slope and the constant *C* can be obtained by regressing *v* against −ln(*ϕ*_1_ − *ϕ*_2_). It gives straight line relationships for most of the cases or for segments of growth or decay. Much better results can be expected, if values of $\frac{d[S]}{t}$ or *dv* are cumulated to give;

26 $$v=\sum _{i=0}^{n}\frac{d[{S}_{i}]}{{t}_{i}}+\cdots \frac{d[{S}_{n}]}{{t}_{n}}=v=\sum _{i=0}^{n}d{v}_{i}+\mathrm{....}\,d{v}_{n}$$

where *i* = number of terms and *n* = last term. This summation is illustrated in Fig. 2(b) and the proof can be stated as;

27 $$({v}_{2}-{v}_{o})-({v}_{1}-{v}_{o})=dv=\frac{[{S}_{2}]-[{S}_{1}]}{({t}_{2}-{t}_{o})}=\frac{d[S]}{t}$$

28 $$\{({v}_{2}-{v}_{o})-({v}_{1}-{v}_{o})\}\,.({t}_{2}-{t}_{o})=[{S}_{2}]-[{S}_{1}]$$

when ${v}_{o},\,{t}_{o}\to 0$, ${v}_{2}-{v}_{1}=\frac{[{S}_{2}]-[{S}_{1}]}{{t}_{2}}$, ${v}_{2}=\frac{[{S}_{2}]-[{S}_{1}]}{{t}_{2}}+{v}_{1}$, where ${v}_{1}=\frac{[{S}_{1}]-[{S}_{o}]}{{t}_{1}}$. Therefore, we computed the experimental *v* values using equation (26) and equation (27) and based on the premise that *dt* remains constant for each cycle during the period of investigation (Tables S2 and S15, Figs S1 and S6).

## Predictions

We can apply the equation (5) to test the hypothesis. Although the results are not very accurate, we can distinguish all of the growth or decay phases. The integral gives much better results, in which;

29 $$P={\int }_{t=0}^{t=n}v\,.dt$$

It can have a straight line relationship or a power function (Table S3), because *P* is embedded in terms of substrate *S* in *v*.

30 $$P=Nvt\pm {P}_{0}$$

31 $$P=N{(vt)}^{q}$$

Lineweaver-Burke plot within a given growth or decay phase yields *v*_*m*_ and *K*_*m*_ values. The overall rate of reaction *v* can then be predicted for expected increases of *S*. The best fit values can be determined by employing equation (30) or equation (31). A computerized program will provide the best result. It is possible to determine the degree of cooperativity in relation to half saturation.

We analysed the two paths *X* and *Y* separately, and believe it to originate from an internal body entity and external food/nutrients supply to determine in each cycle actual *v*_*m*_ and *K*_*m*_ values and thus, the actual generations of enzymes and enzyme substrate complexes.

## Path X - Internal Body Entity

We do not frequently encounter the differential of *v* with respect to [*S*] because *v* is defined as the overall rate constant, and there was no necessity to dwell with rates of changes of *v* and [*S*]. But it is the foundation of our analysis, since *v* = *f*(*S*). Thus, it can be stated as;

32 $$\frac{dv}{d[S]}=\frac{{K}_{m}{v}_{m}}{{({K}_{m}+[S])}^{2}}$$

Therefore, we can find [*S*] by combining quadratic equation (32) with equation (21) to give;

33 $$[S]=\sqrt{{v}_{m}{K}_{m}t}-{K}_{m}$$

We can assume that there are number of MM equations involved in BTs and the “unique” quadratic equation (33) can yield specific ${v^{\prime} }_{m}$ and ${K^{\prime} }_{m}$ values for each of the data points by combining with the differential,

34 $$\frac{d[S]}{dt}=\frac{0.5\,{K^{\prime} }_{m}{v^{\prime} }_{m}}{\sqrt{{K^{\prime} }_{m}{v^{\prime} }_{m}t}}$$

So, we are able to derive both ${v^{\prime} }_{m}$ and ${K^{\prime} }_{m}$ at time *t* (Supplementary Method 3). We validated [*S*] and $\frac{d[S]}{dt}$ precisely with experimental growth data (Tables S4 and S16) applicable to all types of BT reactions. As expected, when we insert ${v^{\prime} }_{m}$ and ${K^{\prime} }_{m}$ values (Tables S4 and S16) into equation (18), we obtain rates of reaction *v*′ for all entities which differ from derived experimental values of *v*. We can attribute this difference to the uniqueness of the quadratic equation, equation (33) (power function), whereas equation (18) is a hyperbolic^48^. We obtain ${k^{\prime} }_{1}$ by rewriting equation (20) as

35 $${k^{\prime} }_{1}={k}_{1}{k^{\prime} }_{m}-{k}_{2}$$

We can derive enzymes and enzyme complexes by using equation (15) to determine $[{E^{\prime} }_{o}]$ and equation (14) to obtain [*ES*′]. Then, using equation (13) we can yield [*E*′] (Tables S5 and S17, Figs S2, S7 and S10). Consequently, we obtained very useful and absolute relationships (Figs S3 and S8), in which

36 $$[E^{\prime} ]=2{[S]}^{0.33}$$

37 $$[E^{\prime} ]=2{[P]}^{0.25}.$$

It seems that it is a substantial and realistic proof upholding an earlier study^14^ because allometric scaling laws in biology are proven by linking *M*^1/4^ and *M*^3/4^ with [*E*′] and *K*_*e*_ in equation (20(a)). It can now be defined as truly enzyme production or generation from body mass, the entity. However, we observed that in some instances or in some cases most times, it is not enough to meet the total requirement, since *v*′ ≠ *v* and *v*′ < *v*. As a result, we analysed the influence of feed and nutrients on the substrate consequently the enzymes.

## Path Y - External Food and Nutrients Supply

By rearranging MM equation (18), *S* = *f*(*v*);

38 $$[S]=\frac{v{K}_{m}}{({v}_{m}-v)}$$

We can deduce the differential of $[S]$ with respect to *v*, $\frac{d[S]}{dv}$, which is equal *t*o *t* according to equation (21), thus we can obtain specific ${v}_{m}^{^{\prime\prime} }$ and ${K}_{m}^{^{\prime\prime} }$ values from;

39 $$\frac{d[S]}{dv}=\frac{{v}_{m}^{^{\prime\prime} }{K}_{m}^{^{\prime\prime} }}{{({v}_{m}^{^{\prime\prime} }-v)}^{2}}=t$$

Then, the reaction rate is,

40 $$v={v}_{m}^{^{\prime\prime} }-\sqrt{\frac{{v}_{m}^{^{\prime\prime} }{K}_{m}^{^{\prime\prime} }}{t}}$$

The differential of;

41 $$\frac{dv}{dt}=\frac{0.5\sqrt{{v^{\prime\prime} }_{m}{K^{\prime\prime} }_{m}}}{{t}^{1.5}}$$

Both ${v}_{m}^{^{\prime\prime} }$ and ${K}_{m}^{^{\prime\prime} }$ at time *t* can be derived from equation (41) (Supplementary Method 4, Tables S6 and S18). We find that, similar to the earlier derivation, the differential and integral can be found precisely. For example, we sought to verify by multiplication of derivatives to determine exact values of $\frac{d[S]}{dt}$ (Tables S7 and S19).

As before, we used equation (15), equation (14), and equation (13) to obtain the amounts of initial enzymes $[{E}_{0}^{^{\prime\prime} }]$, enzyme complexes [*ES*″]_,_ and [*E*″] (Tables S8 and S20, Figs S4, S9 and S11). The derived values were based on *k*_2_ and *k*_1_ values. Notably, ${k}_{1}^{^{\prime\prime} }$ did differ for each application, since derived ${k^{\prime\prime} }_{m}$ were different. In some cases, we find that *v*″ ≠ *v* and *v*″ < *v*. We get values of [*E*′] different to [*E*″] in all cases, because the latter could have been another set of enzymes produced from the nutrient/feed of substrate rather than the enzymes governed by the “body” mass, the entity. Now, we can combine both [*E*′] and [*E*″] as well as [*ES*′] and [*ES*″].

## Combined Function and Inhibitions

**Figure 3:**
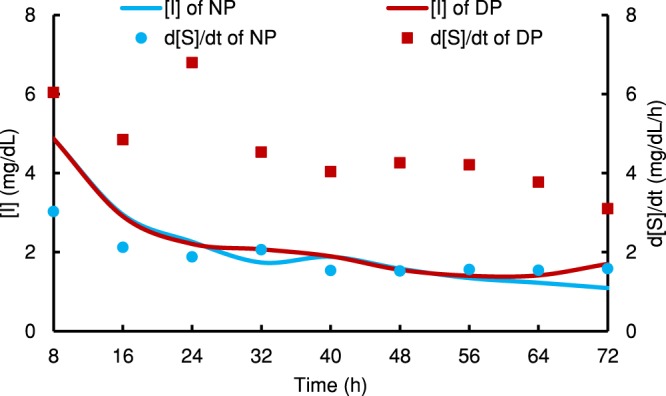
Variations of $\frac{d[S]}{dt}$ (mg/dL/h) and [*I*] (mg/dL) with time of a type 2 diabetes patient (DP) and a normal person (NP).

We can state that the two quadratic functions (equations (33) and (40)) are responsible for the generations of enzymes, whereas, the hyperbolic function (equation (18)) expresses utilization of the enzymes. In other words, the total of enzymes ($[E\prime\prime\prime ]=[E^{\prime} ]+[E^{\prime\prime} ]$) and enzyme complexes ($[ES\prime\prime\prime ]=[ES^{\prime} ]+[ES^{\prime\prime} ]$) thus $[{E\prime\prime\prime }_{0}]=[{E^{\prime} }_{0}]+[{E^{\prime\prime} }_{o}]$ (Tables S9 and S21) are the total generations giving another set of ${v\prime\prime\prime }_{m}$ and ${K\prime\prime\prime }_{m}$, since $v\prime\prime\prime =v^{\prime} +v^{\prime\prime}$. Our investigations in all cases, $v\prime\prime\prime  > v$ at all times, thus manifest inhibitions of the generated enzymes. We believe that the inhibited values of rate of the reaction *v* comprise of both these avenues supplying enzymes and enzyme complexes that were formed. Therefore, we can presume the existence of proportionate values of *v* from *v*′ and *v*″ persisting in the production of enzymes [*E*] and [*ES*] complexes (Tables S10, S11, S22 and S23). Such that;

42 $$v=\alpha v+\beta v$$

where,

43 $$\alpha =\frac{v^{\prime} }{(v^{\prime} +v^{\prime\prime} )}$$

44 $$\beta =\frac{v^{\prime\prime} }{(v^{\prime} +v^{\prime\prime} )}$$

where *α* and *β* values are proportionate values. Therefore, proportionate values of *ES*_*α*_, *E*_*α*_, and *E*_*oα*_ and *ES*_*β*_, *E*_*β*_, and *E*_*o*,*β*_ can be found (Supplementary Method 5). These values when added yield *ES*, *E*, and *E*_*o*_. From these values we are able to deduce *v*_*m*_ from equation (16) and *K*_*m*_ from equation (17). In fact ${K}_{m}={K\prime\prime\prime }_{m}$ making the reactions un-competitive^34^. Therefore, we can deduce inhibitor concentration [*I*] (Supplementary Method 6). It can be illustrated as shown in Fig. 3, where the inhibitions (*I*) for the diabetic patient (*DP*) as opposed to a healthy person (*NP*) are similar, but the blood glucose levels are higher in the diabetic patient.

## Discussion

In this report, we have conceptually developed a cyclic formalism for extending MM kinetics to enhance the validity of their findings, thus expanding the applicability in all BTs. Also the recognition of the uniqueness of rate and time dependency is an important factor of all BTs. It also explains DNA transfer from original substance to the next^37^ even beyond one life cycle. This approach allowed us to comprehensively validate the theory of enzymes being derived from the substrate through biochemical transformation cycles. We were able to hypothetically determine enzymes and enzyme-substrate complexes during the reactions. It is a mathematical model to simulate and predict these inner mechanisms.

We also considered the distinctiveness of $\frac{d[S]}{dt}$ and $\frac{d[S]}{t}$ relationship with the overall reaction rate *v*. In which, any of the changes in the substrate will always be governed by the time lapsed from the commencement of reaction being absolute zero. We realized that, the sensitivity or the accuracy of these results depend on actual time at which the reaction commenced. In other words, it is the ‘time zero’ of reaction. For instance, when we adjust the time to zero in seed germination stage or nursery stage in plants or incubation period of 21 days prior to the birth of a chick, the results are better. We can also verify this time zero by adding an appropriate value to the recorded or experimental time to obtain the best *R*^2^ value for the Lineweaver-Burke plot. Accordingly, we obtained a value of 2.8 days in the case of a dengue fever patient. Perhaps, it implies that the patient might have contracted the virus 2.8 days prior to the reduction of his platelets count.

Such alterations influence the sensitivity of $\frac{1}{{v}_{m}}$ which is the intercept and the slope *K*_*m*_/*v*_*m*_ of the Lineweaver-Burke plot^23^. In fact, the accuracy of these plots is best when *n* of [*S*] = [*P*]^*n*^  approaches ¾. It is noteworthy that the values of *K*_*m*_/*v*_*m*_ are similar among different types of BTs (Table S14) like poultry, plant growth, aerobic composting, and anaerobic digestion. This similarity can only be observed within the same time duration because of time dependency. This ratio ${K}_{m}/{v}_{m}$ signifies inhibitions. If this ratio is low, it is a preferred result among the classification of BTs.

The most commonly used methods of Lineweaver-Burke plot and Eadie-Hofstee plots are approximations to determine *v*_*m*_ and *K*_*m*_ values, whereas combined equation gives actual *v*_*m*_ and *K*_*m*_ values for each and every data point. This allows determining types of inhibitions^34^ in relation to maximum *v*_*m*_ value and lowest *K*_*m*_ value. We made a comparison between these approximations and average values from the combined equation obtained from our analysis (Fig. S5, Tables S13 and S14). Accuracy is less when using these average values in combined equation. Therefore, numerical methods and non-linear regression models^18,23,49,50^ can be used to predict accurately *v* for an assumed value of *S*.

Sometimes all of these outcomes depend on the data acquisition process and method of analysis. For example, the platelet count fluctuates during the day of any human. The count taken at specific time intervals, for each day can be considered as a differential value, which could be used to compare dynamics of platelets counts. It applies also to wastewater treatment systems in which a complete cycle of microbial activity can be better understood where there are distinct growth and decay phases distinguishable in the analysis^22,51^. If we sum-up values of VSS in wastewater treatment, the effect would not have a physical entity at all, but could represent idealistic growth. Such a value can be obtained, if we take samples and analyse for VSS from inlet to outlet along the length of a wastewater treatment system, in which treatment time is proportional to the length. In plants, the growth and decay of roots and root hairs, including storage of nutrients, energy, and precipitation of substances were not considered in the analysis. Only the plant height was taken as a measure of growth, where we can visualize an actual increment in the physical entity, but may not be the absolute amount. We have developed an analytical tool which can rely on data obtained with least interventions and non- destructive methods, as well as less time consuming, since we can obtain BTs of plants identical to the growth of a chicken. Let us assume that we can record an event in the past like a drought period or diseases of a substance. We should be in a position to describe such influential behaviour of this substance in the present and future times. We can only express such phenomena mathematically by cumulating the values of substances over defined time durations.

All BTs undergo both growth and decay. In decay, enzymes or microbial enzymes both types form products. As we have viewed in this analysis, like in the growth phase, the decay phase also provides enzymes and substrates from biomass to form enzyme-substrate complexes before converting to solids, liquids, and gases. The enzyme formations are governed by temperature, pH, and ionic strength. Therefore, different types and amounts of enzymes are formed from one cycle to the next. For example, VS content will decrease in a decay process while the product, CO_2_ will increase. The reduced amount of *d*[*VS*] is proportional to CO_2_ productions, thus cumulative of *d*[*VS*] describe total generations of products, namely CO_2_ in this case.

**Figure 4:**
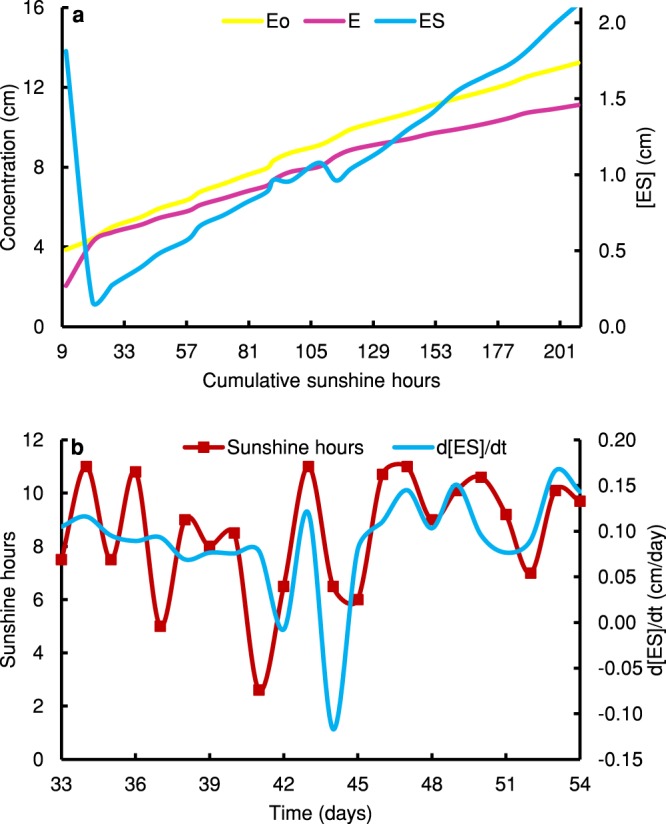
Variations of [*E*_*o*_], [*E*], and [*ES*] in a tomato trial (**a**) [*E*_*o*_], [*E*], and [*ES*] in response to cumulative sunshine hours (**b**) Sunshine hours and $\frac{d[ES]}{dt}$ variations with time. It was assumed that cumulative plant height is proportionate to plant weight.

Cumulative effects can be associated with any biological life processes and activities and these accumulations will influence future times of its existence. We can prove this concept by analysing the tomato trial, where we observed [*ES*] to have a direct relationship to environmental factors, such as the number of sunshine hours accumulated throughout the growth (Fig. 4(a)). However, we observed that enzyme levels were much higher in comparison to low levels of [*ES*]. Therefore, we can decide on the limiting factor to be the formation of [*ES*]. We also can distinguish the transition of growth phases which is very noticeable in Fig. 4(b). In fact, we see in the second phase, increases in $\frac{d[ES]}{dt}$ with time, responding much more to longer duration of sunshine hours. We can distinctly identify [*ES*] at the beginning to be high and it might be attributed to complexes formed during the period before transplanting.

In the case of the hen, actual growth is known, yet high [*ES*] at commencement can be observed in the egg, because of rapid growth after hatching. Notably, body weight *M*, [*S*], *v*, $v\prime\prime\prime$, [*E*], and $[E\prime\prime\prime ]$ are related to cumulative feed intake as power functions, perhaps showing differences in energy levels^15^. The generations of enzymes and formation of enzyme-substrate complexes are governed by the two well defined quadratic equations, giving rise to large quantities of enzymes and enzyme-substrate complexes that are expressed as hyperbolic functions $v\prime\prime\prime =\frac{{{v}^{\prime\prime\prime }}_{m}[S]}{{K\prime\prime\prime }_{m}+[S]}$, which is in fact the highest level in relation to *v*. The lower level is then *v*, which is the transformed value, thus expressing $v=\frac{{v}_{m}[S]}{{K}_{m}+[S]}$. It should be noted that $[ES\prime\prime\prime ] > [ES]$ and $[E\prime\prime\prime ] > [E]$. The actual inhibitions to growth may arise from the prevailing environmental conditions as well as supplying energy for metabolic activities, including respiration, which involves the generation and utilization of enzymes^52–54^. On the 85^th^ day in the hen, the higher level $v\prime\prime\prime$ corresponds to energy content of cumulative feed intake of 90 MJ^55^. Out of which 19.9 MJ conserved in mammalian tissue^15^ at the lower level *v*.

We can also ascertain a host of information on both the growth and decay which are taking place simultaneously in different BT reactions like in the cases of bioreactors and diabetic patients. In our analysis, we did not encounter negative values in [*S*], $\frac{d[S]}{dt}$, [*E*], *k*_1_, and *k*_2_, except in the case of diabetic patient of -[*E*], but did surface negative values as outcomes in *v*_*m*_, *K*_*m*_, all forms of [*E*_*o*_], [*ES*], $\frac{d[E]}{dt}$, $\frac{d[ES]}{dt}$, ${k^{\prime} }_{1}$ values as well as in inhibitions, $\frac{[I]}{{k}_{i}}$, of a diabetic case (Fig. S12). Such manifestation of $-\,\frac{d[ES^{\prime} ]}{dt}$ in the body of the Caucasian regaining weight in a dieting regime can mean much less enzymes production, corresponding to the loss in weight (Fig. S13). In fact, in all these cases, much better and useful interpretations can be made by medical practitioners. In the case of the tomato trial, an agronomist will be able to extract information on solar incidence influencing growth. If we consider solar incidence, nutrient availability, and other variables including microbial activity, we will certainly get better and more useful results. Therefore, vigilant monitoring of these parameters preferably for defined time periods and possible interventions will bring about expected results. The on-going BT processes could be analysed, evaluated, interpreted, and viewed in all the derivations. Therefore, it is a diagnostic tool to examine inner mechanisms of BT for academia, researchers, and professionals. It is also a guide for technicians and lay persons for day to day use.

We believe this study to be of value because it expresses mathematically all biological materials governed by well-defined BT kinetics. It is clear, the inner mechanisms of a single parameter can be examined and evaluated in depth and also compared with other parameters governing the functions of any given entity.

## Electronic supplementary material

Supplementary information
