# Supplementary material for: Extensions to Michaelis-Menten Kinetics for Single Parameters
Source: Sci Rep. 2018 Nov 8;8:16586. doi: 10.1038/s41598-018-34675-2 (PMC6224567; doi:10.1038/s41598-018-34675-2)
Supplement: Supplementary file 1 — Supplementary information [file 41598_2018_34675_MOESM1_ESM.pdf]

Supplementary Information for

Extensions to Michaelis-Menten Kinetics for Single Parameters

R. T. K. Ariyawansha, B. F. A. Basnayake*, A. K. Karunarathna, M. I. M. Mowjood

*Correspondence to: benb@pdn.ac.lk

This PDF file includes:

Supplementary Methods (1-6)
Supplementary Text: Definition of the reactants
Supplementary Figures (Fig.S1- S13)
Supplementary Tables (Table S1- Table 23)

Supplementary Methods

1. Data acquisition for the analysis
2. Derivation of k_2
3. Derivation of $v_m^{'}$ and $K_m^{'}$ at time *t*
4. Derivation of $v_m^{''}$ and $K_m^{''}$ at time *t*
5. Calculation of proportionate values of enzymes and enzyme-substrate
6. Calculation of inhibitor concentration $\left[I\right]$

Supplementary Figures (Fig.S1- S13)

Figure S1. Illustration of rate of reaction *v* as a function of substrate in growth of a hen
Figure S2. The generation of $\left[E^{'}\right]$in relation to substrate $\left[S\right]$ of body entity in a hen growth
Figure S3. Relationship between $\left[P\right]$ and $\left[E^{'}\right]$, $\left[S\right]$ and $\left[E^{'}\right]$ of a hen growth
Figure S4. The generation of enzymes $\left[E^{''}\right]$ from the substrate governing the reaction of a hen growth
Figure S5. Determination v_m and K_m of a hen growth data (**a**) Lineweaver-Burke plot (**b**) Eadie-Hofstee plot
Figure S6. Illustration of rate of reaction *v* as a function of substrate of *Amaranthus oleraceus* plant growth (cumulative height (cm) $\propto$ weight (g)) experiment
Figure S7. The generation of $\left[E^{'}\right]$in relation to substrate $\left[S\right]$ of body entity in *Amaranthus oleraceus* plant growth
Figure S8. Relationship between $\left[P\right]$ and $\left[E^{'}\right]$ *and* $\left[S\right]$ and $\left[E^{'}\right]$*Aamaranthus oleraceus* plant growth (cumulative height (cm) α weight (g)) (a.) $\left[P\right]$ and $\left[E^{'}\right]$ (b.) $\left[S\right]$ and $\left[E^{'}\right]$
Figure S9. The generation of enzymes $\left[E^{''}\right]$ from the substrate governing the reaction in *Amaranthus oleraceus* plant growth
Figure S10. The generation of $\left[E^{'}\right]$in relation to substrate $\left[S\right]$ of body entity in different experiments

Figure S11. The generation of enzymes $\left[E^{''}\right]$ from the substrate governing the reaction in different experiments

Figure S12. Rates of change of variations of substrate concentrations $\frac{d[S]}{dt}$, enzyme concentrations ($\frac{d[E]}{dt}$), enzyme substrate concentrations ($\frac{d[ES]}{dt}$) time of a type 2 diabetes patient (female) with time

Figure S13. Weight changes of a Caucasian, regaining weight in the dieting regime

Supplementary Tables (Table S1- Table 23)

Table S1. $[S]/[P]$ ratio of different experiments influenced by microbial populations

Table S2. The calculation to derive *v* from body weight of a hen

Table S3. Comparison of prediction of *v* for different experiments

Table S4. Derivation of $K_m^{'}$ and $v_m^{'}$ for simulating substrate [S] of a hen growth

Table S5. Derivation of $\left[E^{'}\right]$ generation from body entity in relation to $[S]$ from experimental data of a hen growth

Table S6. Derivation of $K_m^{''}$ and $v_m^{''}$ for simulating reaction rate (*v*) of a hen growth

Table S7. Verification of $\frac{d[S]}{dt}$ with the multiplication of derivatives of a hen growth data

Table S8. Derivation of $\left[E^{''}\right]$ generation from the substrate governing the reaction of a hen growth

Table S9. Calculation of $\left[E_o^{'''}\right]$, $\left[ES^{'''}\right]$, and $\left[E^{'''}\right]$ of growth of a hen

Table S10. Calculation of $[ES_\alpha]$, $[E_\alpha]$, $[ES_\beta]$, and $[E_\beta]$ of a hen growth

Table S11. Calculation of $v_{m(\alpha+\beta)}$, $K_{m(\alpha+\beta)}$, and v of a hen growth

Table S12. Variations in average v_m and K_m values simulated from combined equation and Lineweaver-Burke and Eadie-Hofstee plots for the case of a hen's growth

Table S13. Comparison of v deduced from Lineweaver-Burke plot (LBP), Eadie-Hofstee plot (EHP) and Combined Equation (CE)

Table S14. Comparison of K_m/v_m deduced from Lineweaver-Burke plot (LBP), Eadie-Hofstee plot (EHP) and Combined Equation (CE) in different experiments

Table S15. The calculation to derive *v* from *Amaranthus oleraceus* plant growth (experimental data)

Table S16. Derivation of $K_m^{'}$ and $v_m^{'}$ for simulating [S] of *Amaranthus oleraceus* plant growth

Table S17. Derivation of $\left[E^{'}\right]$ generation from body entity in relation to $[S]$ from experimental data of *Amaranthus oleraceus* plant growth

Table S18. Derivation of $K_m^{''}$ and $v_m^{''}$ for simulating reaction rate (*v*) of the experimental data in *Amaranthus oleraceus* plant growth

Table S19. Verification of $\frac{d[S]}{dt}$ with the multiplication of derivatives of *Amaranthus oleraceus* plant growth experiment

Table S20. Derivation of $\left[E^{''}\right]$ generation from the substrate governing the reaction of *Amaranthus oleraceus* plant growth experiment

Table S21. Calculation of $\left[E_o'''\right]$, $\left[ES'''\right]$, and $\left[E'''\right]$ of *Amaranthus oleraceus* plants growth

Table S22. Calculation of $\left[ES_\alpha\right]$, $\left[E_\alpha\right]$, $\left[ES_\beta\right]$, and $\left[E_\beta\right]$ of *Amaranthus oleraceus* plant growth

Table S23. Calculation of $v_{m(\alpha+\beta)}$, $K_{m(\alpha+\beta)}$, and v of *Amaranthus oleraceus* plant growth

Supplementary method 01
Data acquisition for the analysis
The existing data based on research conducted by Solid Waste Management Research Unit, Faculty of Agriculture, University of Peradeniya was gathered and compiled to allow detail analysis, synthesis for developing the desired mathematical expressions. Brief descriptions of the experimental procedures of the used experimental data for our kinetic analysis are given in this section. We used already published data (Ahmad et al., 2011[*]) on platelets count variations of dengue patient before and after treatment of *Carica papaya* leaves extract We have obtained the consents to use the other data related to the investigation on humans. In the case of study on diabetes, approval was given for the study from the ethical clearance of Faculty of Allied Health Sciences, University of Peradeniya. Mr. Jeremy Burgmen has given his consent to use his body weight data. In relation to animals, we have used already published data (*55*) on the growth of chicken in relation to feed rations given to those animals.
Note:
*Ahmad, N. et al. Dengue fever treatment with Carica papaya leaves extracts. Asian. Pac. J. Trop. Biomed. 1(4), 330-333 (2011).

1. Evaluation of Solar Aeration Chimney composting system with pre-sorted municipal solid waste

This study entailed design and evaluation of a new composting system, which consists of low-cost aeration system known as "Solar Aeration Chimney". The system consists of three parts namely, solar capturing panel, chimney, and pipe system. The designed structure was filled with pre-sorted municipal solid waste. All the physical and chemical parameters related to the composting process were evaluated daily for 25 days. Three replicate samples were used to determine the average value. The volatile solids (VS) % (w/w) of the decomposing pile at time *t* throughout the experimental period was used for this study. Cumulative volatile solids % (w/w) at time *t* for 25 days was calculated and it was considered as *P* for the study.

2. Production of bio-char fertilizer by using Solar Aeration Chimney composting system

This research was conducted to produce super bio-char fertilizer by using the Solar Aeration Chimney composting system. The chimney was constructed by using zinc coated GI sheet. Then chimney and chimney support were painted by black color with anticorrosive paint to increase heat absorption. The chimney height was 7 m and diameter was 38 cm. First, the chimney support was fitted to the concrete pad and then chimney was erected and fixed. The piping system was constructed of PVC. The function of the piping system is to suck the air through waste pile and the diameter of the pipe is 100 mm. There were two riser pipes with end caps, which were 75 cm in height and it consisted of slots to enable sucking of the air through the waste pile. Then this PVC pipe system was connected to the chimney. This system has a valve to remove condensed water and leachate. The designed and fabricated structure was filled with a mixture of shredded market waste, bio-char, and Eppawela Rock Phosphate (ERP). Bio-char was size reduced to less than 2.5 cm for the experiment. The mixing ratio of market waste to bio-char was 3.5:1 on the dry weight basis. Therefore, 200 g of ERP was added for 3,108 kg of the total amount of waste pile. The evaluation and monitoring were done for 60 days. Sampling was done daily from the top, middle, and bottom layers of the pile. Collected samples were analyzed for the moisture content, total solid % (w/w), VS % (w/w), and ash content % (w/w). VS % (w/w) of the mid stratum variations during the study period was used for this analysis. Cumulative VS % (w/w) at time *t* for 60 days was calculated and it was considered as *P* for the study.

3. Evaluation of wet and dry layered odour controlling bio-filter

Biofilter setup consisted of two cylindrical filter beds which can be removed. It had a plenum chamber that had a conical shape to ensure uniform air distribution through the filter beds. The thickness of the layers was 10 cm and a 10 cm gap was kept between these filter beds. An average moisture content of upper layer was maintained at 31 % (w/w) on the wet basis and the lower layer was maintained on average of 39.5 % (w/w). The filter bed characteristics were evaluated at three days intervals for first 163 days of operation. Thereafter, sampling was done once a week. Samples were taken for evaluating the filter media so as to control pH, moisture content, temperature, VS % (w/w), and total nitrogen content. Humidification was done after 106 days of operation. The relative humidity and temperature of the biofilter gas stream were measured inside the plenum chamber, in between, upper, and lower filter beds and just above the upper filter bed by using a portable relative humidity meter. The VS % (w/w) of the lower biofilter layer at time t throughout the experimental period was used for this study. Cumulative VS % (w/w) at time t for 303 days was calculated and it was considered as P for the study.

4. Anaerobic hydrolytic reactor for kitchen waste treatment

A hydrolysis phase column type anaerobic reactor study was conducted at the Mewathura Farm, Faculty of Agriculture, University of Peradeniya. The reactor was fabricated by using a 150 mm diameter PVC pipe with a volume of 21.8 L. The outlet of the reactor ended up with a rubber tube and it was closed using a wooden stopper. This study was directed initially to develop the hydrolysis phase of the two-phase anaerobic digestion process. Characteristics of elute and the digested solids were analyzed to determine the optimum operating conditions of experimental runs for the processes. The reactor was filled with 1.5 kg kitchen waste and 3.75 L fresh water in one day's feeding. The reactor was fed for 3 days. The volatile suspended solids (VSS) concentration (mg/L) of elute of the anaerobic reactor at time t throughout the experimental period was used for this kinetic analysis. 20 % of VSS is considered to be the population of microbial solid concentration (*1*). Cumulative VSS concentration (mg/L) at time t for 30 days was calculated and it was considered as P for the study.

5. Evaluation of typical weekly body weight changes with time of a hen

Typical weekly body weight and feed requirements of a hen fed with well-balanced diets providing 3,200 kcal /kg (1,452 kcal /lb) from 1st week to 9th week as reported by Jacob and co-workers (*55*) were used for the analysis. The initial time taken for the egg to hatch was assumed to be 22 days. The average weight of a one day old chick was assumed to be 40 g and it was incorporated to the data set of Jacob and co-workers (*55*). Body weight at time t was considered as P for the study.

6. Evaluation of typical weekly body weight changes with time of a male broiler chicken

Similarly, typical weekly body weight and feed requirements of a male broiler chicken fed with well-balanced diets providing 3,200 kcal/kg (1,452 kcal/lb) from 1st week to 9th week as reported by Jacob and co-workers (*55*) were used for the analysis. The initial time taken for the egg to hatch was assumed to be 22 days. The average weight of a one day old chick was assumed to be 40 g and it was incorporated to the data set of Jacob and co-workers (*55*). Body weight at time t was considered as P for the study.

7. Evaluation of the effects of biochar application on *Amaranthus oleraceus* plant growth

The field experiment was conducted in the Coconut Cultivation Board in Madamulana, Sri Lanka. It belongs to Hambanthota district and dry zone low country. Charcoal was produced

from coconut shells by using pit method. The particle size of the produced charcoal was reduced and these materials were sieved by using 5 mm size sieve. *Amaranthus oleraceus* was selected as the planting material. 0.7 m x 2.15 m size planting bed was prepared. The soil conditions of the planting bed were analyzed for pH, moisture content, ash, total dissolved solids (TDS), electrical conductivity (EC), total nitrogen, phosphorus, and potassium, before planting, 10 days and 20 days after planting. Each plant height was measured firstly 10 days of planting and thereafter ones in two days for 30 days. The average height of the plants at time *t* throughout the experimental period was used for this study. Cumulative height (cm) at time *t* was calculated and it was considered as *P* for the study.

8. Evaluation of blood glucose variations of type 2 diabetes patients

This preliminary study was conducted to develop a mathematical model expressing enzyme kinetics for the interpretation of selected parameters and data, so as to validate the developed mathematical model in type 2 diabetes. The ethical clearance was obtained from the Ethical Committee of Faculty of Allied Health Sciences, University of Peradeniya. Thereafter, the permission from the written consents of diabetic patients was obtained after explaining the objectives of the research project. Nursing history was taken from each patient according to history format. A female patient of 58 years old who had been admitted to the Diyathalawa Base hospital during 18th - 25th December 2013 was selected as patient 01. Morning, noon, night values of capillary blood sugar (CBS) rates, fasting blood sugar (FBS) levels, morning and noon sugar color, full blood count (FBC) values intake and output of the patient 01 were obtained from the records during her treatment in the hospital for three days. Cumulative CBS (mg/dL) at time *t* was calculated and it was considered as *P* for the study.

And also, a 68 years old female patient who was diagnosed with chronic kidney disease, had been admitted to the Diyathalawa Base hospital medical ward and intensive care unit during the period of 21st - 31st January 2014 was selected as patient 02. Morning, noon, night values of CBS rates, FBC values, blood urea levels were obtained from the records during her treatment in the hospital for ten days. Cumulative CBS (mg/dL) at time *t* was calculated and it was considered as *P* for the study.

9. Platelets count variations of dengue patient before and after treatment of *Carica papaya* leaves extract (Ahmad et al., 2011)

The data were collected through secondary data analysis. The performed secondary analysis dataset was obtained from journal article titled "Dengue fever treatment with *C. papaya* leaves extracts" which was published by Ahmad and co-workers in 2011. Written permission from the corresponding author of this article was obtained through e-mail conversation. As reported by Ahmad and co-workers (Ahmad et al., 2011) blood samples were obtained from a dengue patient in 24 hours intervals for five consecutive days and evaluated for biochemical parameters of platelets count, white blood cells (WBC), Red Blood Cells (RBC), and Neutrophils (NEUT). After that, as a treatment, *C. papaya* leaves extract was prepared in water and 25 mL of aqueous extract was administrated to the patient twice daily for five consecutive days. Blood samples were obtained from the patient in 24 hours intervals during this period and evaluated for the platelets count, WBC, RBC, and NEUT (Ahmad et al., 2011). The platelet count x E+03/dL variations before and after administration of *C. papaya* extract was used separately for this kinetic analysis. Cumulative platelet count x E+03/dL at time *t* was calculated and it was considered as *P* for the study.

10. Variation of biochemical oxygen demand (BOD) of dumpsite leachate

A leachate sample was taken from Gohagoda dumpsite, Sri Lanka and analysis was done at the University of Peradeniya. Initial quality of the samples was tested for pH, conductivity,

salinity, total dissolved solids (TDS), total suspended solids (TSS), volatile suspended solids (VSS), total solids (TS), volatile solids (VS), and chemical oxygen demand (COD). The BOD of the sample was determined through Winkler titration method on daily basis for six days with 75 % dilution level. BOD (mg/L) values at time *t* were used for this kinetic analysis. Cumulative BOD (mg/L) at time *t* was calculated and it was considered as *P* for the study.

11. Evaluation of an up flow anaerobic sludge blanket reactor performances
An up-flow anaerobic sludge blanket (UASB) reactor was designed and fabricated to treat food and fruit wastes that were generated daily in a *Cucumis sativus* fruits processing factory. The reactor consisted of three major components namely feeding system, main reactor, and gas collecting system. De-brined gherkin slices were collected from the slicing section of the company. Papaya and pineapple wastes were collected from the organic section. Papaya waste consisted of peel and papaya paste. In the sorting section, damaged gherkin fruits were collected. In addition to that, some kitchen and food waste were collected from the company canteen. All of these wastes were collected according to a predetermined ratio. Before feeding, the wastes were cut into very small particles, especially gherkin pieces. Waste to water ratio was taken as 1:2 (wet basis). Daily feeding of the reactor was done. But in the first stage of the feeding, gherkin to food waste ratio was kept at 1:1 to facilitate anaerobic reactions. Salt (salinity) is not a setback when adhering to the predetermined feeding ratio. To evaluate the performance of the reactor, samples were taken from the top, bottom and middle two sampling points. Collected samples were analyzed for TDS daily for 30 days. TDS (mg/L) values of the samples taken from the middle of the reactor at time *t* were used for this kinetic analysis. Cumulative TDS (mg/L) at time *t* was calculated and it was considered as *P* for the study.

12. Evaluation of *Cucumis sativus* growth with biochar application
This research was conducted at the University of Peradeniya, Sri Lanka with the collaboration and the supervision of HJS Condiments Limited. The experimental land was prepared using a disk harrow mounted to the four-wheel tractor and mammoties. And raised beds were prepared with a length of 4.5 m, a width of 0.9 m and a height of 0.23 m. The spacing between two beds was 0.23 m. Ten treatments with four replicates were arranged in a randomized complete block design (RCBD). One of the treatments was 70 % biochar + 30 % inorganic fertilizer of recommended quantity and compost (T9). To evaluate the biochar, soil quality, and fertilizer/biochar amended soil, samples were taken, before planting, 15 days after crop establishment and these samples were analyzed for the soil parameters. Each vine length was measured firstly after 7 days of seeding and thereafter daily for 25 days. The average vine length at time *t* throughout the experimental period of block four of T9 was used for this kinetic analysis. Cumulative vine length (cm) at time *t* was calculated and it was considered as *P* for the study.

13. Evaluation of effects of a developed fertilizer on *Solanum lycopersicum* plant growth
The research was conducted at the Department of Agricultural Engineering, Faculty of Agriculture, University of Peradeniya, Peradeniya to determine soil stability with a developed organic fertilizer (DOF) by the University of Peradeniya and evaluated the effect of the fertilizer on *Solanum lycopersicum* plant growth. Characteristics of DOF, compost, and soil were analyzed. Four treatments were applied. One of the treatments was 70 % of DOF + 30 % of recommended fertilizer (RF) as fertilizer (T3). After 30 days of seeding, the crop was established. Each plant height was recorded daily. And also, daily sunshine hours were measured by using a Campbell-Stokes sunshine recorder. The average plant height of T3 at

time *t* throughout the experimental period was used for this kinetic analysis. Cumulative plant height (cm) at time *t* was calculated and it was considered as *P* for the study.

14. Caucasian weight

A 57 year old male having an average weight of 99 kg body weight was recorded by himself for 31 days daily at the same time of the day. He had recorded the weight during a dieting program that he underwent. The written consent of the individual was obtained after explaining the objectives of the research. Cumulative weight (kg) at time *t* was calculated and it was considered as *P* for the study.

Table S1.
$[S]/[P]$ ratio of different experiments influenced by microbial populations

Substrate	$[S]/[P]$	Description
Volatile solids (%)	0.21	A low cost aeration system which is known as Solar Aeration Chimney composting system was filled with biodegradable fraction of municipal solid waste. Sampling was done daily for 25 days to determine the decomposition process.
	0.17	A low cost aeration system known as Solar Aeration Chimney composting system was filled with a mixture of market waste, bio-char, and Eppawela rock phosphate. Sampling was done daily for 60 days to determine the decomposition process.
	0.21	Bio-filter media of wet matured compost: Odour controlling bio-filter which consisted of wet compost was evaluated for 303 days.
Volatile suspended solids (mg/L)	0.21	An anaerobic hydrolytic reactor: A hydrolysis phase reactor was fed with kitchen waste to water ratio was 1 kg : 2.7 L respectively. Sampling was done daily for 30 days to determine the decomposition process.

Note: Experimental procedures of primary data collections are given in **Supplementary method 1**(data acquisition for the analysis).

Supplementary Text – Definition of the reactants

The term *substrate* has another meaning in chemistry. Some chemical syntheses are carried out in mixed phases; for example, the *reactants* exist in solution but the reaction itself occurs at the surface of a solid. The identity of the solid, specified in experimental protocols, influences the **synthesis** reactions, and the solid is referred to as the substrate.

Read more: http://www.chemistryexplained.com/St-Te/Substrate.html#ixzz5Poyc91PV

Supplementary method 2

Derivation of k_2

The mass action law applied to the resultant cycle *D* to *E* to *C* to *D* of equation (6) can be written as equation (19).

$$\frac{d[P]}{dt} = -k_2[P][E] + k_2[ES] + k_1[E][S] \qquad (19)$$

At equilibrium, $\frac{dP}{dt} = 0$

It leads to the solution of equation (20) as mentioned below.

$$k_1[E][S] = -k_2[P][E] + k_2[ES] \qquad (S1)$$

By substituting, $k_2[ES] = k_1[E][S] - k_1^{'}[ES]$ to equation (S1),

$$0 = -k_2[P][E] + k_1[E][S] - k_1^{'}[ES] + k_1[E][S] \qquad (S2)$$

And then equation (S2) dividing by $k_2[P][E]$,

$$0 = \frac{-k_2[P][E]}{k_2[P][E]} + \frac{2k_1[E][S]}{k_2[P][E]} - \frac{k_1^{'}[ES]}{k_2[P][E]} \qquad (S3)$$

In all cases of experimental data analysis, $1 \approx \frac{k_1^{'}[ES]}{k_2[P][E]}$

$$0 = -1 + \frac{2k_1[S]}{k_2[P]} - 1 \qquad (S4)$$

$$2 = \frac{2k_1[S]}{k_2[P]} \qquad (S5)$$

Therefore, $k_2 = \frac{k_1[S]}{[P]}$ (20)

Application of developed formulism to experimental data
Example 01: Evaluation of typical weekly body weight changes with time of a hen

Typical weekly body weight and feed requirements of a hen fed with well-balanced diets providing 3,200 kcal /kg (1,452 kcal /lb) from 1st week to 9th week as reported by Jacob and co-workers (*55*) were used for the analysis. The initial time taken for the egg to hatch was assumed to be 22 days. The average weight of a one day old chick was assumed to be 40 g and it was incorporated to the data set of Jacob and co-workers (*55*). Body weight at time *t* was considered as *P* for the study.

The calculation to derive reaction rate *v* by using equation (21) from body weight of a hen is given in **Table S2** and **Fig. S1**.

Table S2.
The calculation to derive *v* from body weight of a hen

Time (days)	Body weight (*P*) (g)	$\frac{d[P]}{dt}$ (g/day)	$[S]=[P]^n$ $n = 0.75$ (g)	$d[S]$ (g)	$\frac{d[S]}{t}$ (g/day)	$v = \sum\frac{d[S]}{t}$ (g/day)
22*	40**	1.82	15.91	15.91	0.72	0.72
29	145	15.00	41.79	25.88	0.89	1.62
36	345	28.57	80.05	38.27	1.06	2.68
43	617	38.86	123.80	43.75	1.02	3.70
50	966	49.86	173.27	49.48	0.99	4.69
57	1,343	53.86	221.85	48.57	0.85	5.54
64	1,742	57.00	269.64	47.79	0.75	6.28
71	2,132	55.71	313.75	44.11	0.62	6.91
78	2,504	53.14	353.98	40.22	0.52	7.42
85	2,844	48.57	389.45	35.47	0.42	7.84

Note : *The initial time taken for the egg to hatch was assumed to be 22 days.
**Average weight of a one day old chick was assumed to be 40 g.

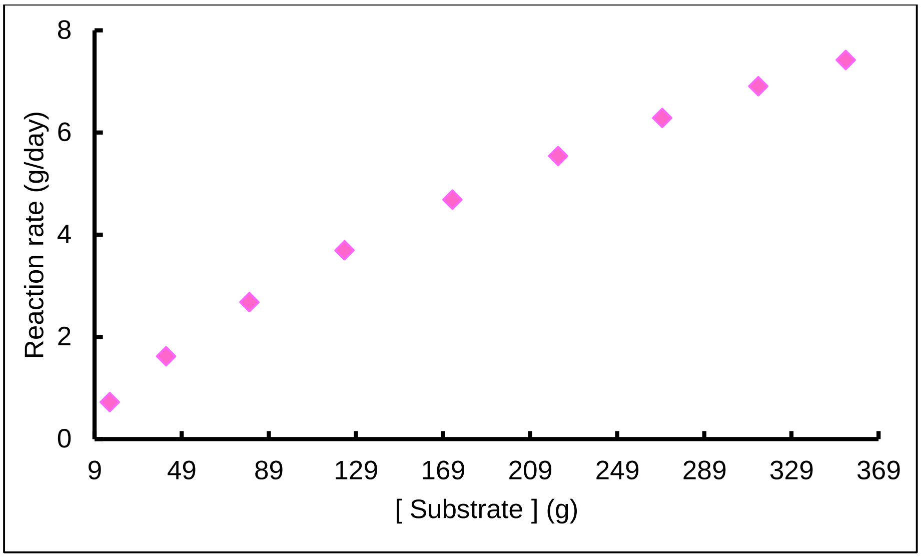

Figure S1.
Illustration of rate of reaction *v* as a function of substrate in growth of a hen

Prediction of *v*

A straight line relationship of $P = 3.0866vt - 39.421$ with R^2 value of 0.996 and a power function $P = 2.2627(vt)^{1.0611}$ with R^2 value of 0.995 obtained from equation (30) and equation (31) for hen's experimental data. Where, P = product, v = reaction rate, t = time

Table S3.
Comparison of Prediction of *v* for different experiments

Experiment	Linear	R^2	Power	R^2
A hen's growth (*55*) : Weight changes in g from 22nd day to 85th day of age	$P = 3.0866vt - 39.421$	0.996	$P = 2.2627(vt)^{1.0611}$	0.995
Male-chick's growth (*55*): Weight changes in g from 22nd day to 85th day of age	$P = 3.388vt + 44.593$	0.992	$P = 2.086(vt)^{1.0874}$	0.996
Amaranthu soleraceus plant growth: plant height in cm as the growth parameter from 10th to 30th day after seedling emerged.	$P = 2.2096vt - 4.6363$	0.999		
Cucumis sativus vine growth: vine length in cm as the growth parameter from 10th to 30th day after seedling emerged.	$P = 3.5503vt - 39.494$	0.984	$P = 0.8692(vt)^{1.2508}$	0.997
Solanum lycopersicum plant growth: plant height in cm as the growth parameter from 31st to 54th day after seedling emerged.	$P = 4.3766vt - 49.761$	0.995	$P = 1.1959(vt)^{1.2445}$	1
Blood glucose levels (mg/dL): blood glucose levels of a type 2 diabetes female patient were measured at 8 hour intervals for 72 hours.	$P = 2.0927vt - 149.21$	0.995	$P = 6.5919(vt)^{0.8423}$	0.999
Platelets count (E+03/dL) variations of a dengue patient after treatment of *Carica papaya* leaves extract were measured daily for 5 days (Ahmad et al., 2011).	$P = 2.1673vt + 23.144$	1	$P = 4.1142(vt)^{0.8898}$	1
Variations of daily biochemical oxygen demand (BOD_1-BOD_6) (mg/L) of a dumpsite leachate sample were measured for 6 days.	$P = 3.1606vt + 15.644$	1		
A Solar Aeration Chimney composting system was filled with a mixture of market waste, bio-char and Eppawela Rock Phosphate (ERP). VS % (w/w) variations of the mid stratum of the decomposing pile were analyzed daily for 60 days.	$P = 0.9013vt + 175.67$	0.998	$P = 4.3854(vt)^{0.8054}$	0.998
Total dissolved solids (mg/L) concentrations in an up-flow anaerobic sludge blanket reactor were measured daily for 30 days.	$P = 2.3639vt + 1035.7$	0.999	$P = 11.567(vt)^{0.8287}$	0.997

Note: Experimental procedures of primary data collection are given in **Supplementary method 1**(data acquisition for the analysis)

Analysis of Path X - Internal Body Entity

Supplementary method 3
Derivation of $v_m^{'}$ and $K_m^{'}$ at time *t*

We are able to derive both $v_m^{'}$ and $K_m^{'}$ at time *t* from the following equations which derived by rearranging equation (34) as given below (**Table S4**).

$$\frac{d[S]}{dt} = \frac{0.5K_m^{'}v_m^{'}}{\sqrt{K_m^{'}v_m^{'}t}} \quad (34)$$

$$K_m^{'} = \left(\frac{d[S]}{dt} \times \frac{t}{0.5}\right) - [S] \quad (S6)$$

$$v_m^{'} = \frac{t}{\left(\frac{0.5}{d[S]/dt}\right)^2 . K_m^{'}} \quad (S7)$$

We validated $[S]$ and $\frac{d[S]}{dt}$ precisely with experimental growth data as given in **Table S4**.

Table S4.
Derivation of $K_m^{'}$ and $v_m^{'}$ for simulating substrate [S] of a hen growth

Time (days)	[S] (g)	$K_m^{'}$ (g)	$v_m^{'}$ (g/day)	$[S]=\sqrt{v_m K_m t}-K_m$ (g)	$\frac{d[S]}{dt}=\frac{0.5K_m^{'}v_m^{'}}{\sqrt{K_m^{'}v_m^{'}t}}$	$v^{'}=\frac{v_m^{'}[S]}{K_m^{'}+[S]}$ (g/day)
22	15.9	15.9	2.9	15.9	0.72	1.45
29	41.8	172.9	9.2	41.8	3.70	1.79
36	80.0	312.8	13.7	80.0	5.46	2.79
43	123.8	414.1	16.2	123.8	6.25	3.74
50	173.3	534.0	18.7	173.3	7.07	4.59
57	221.8	568.2	19.3	221.8	6.93	5.41
64	269.6	604.7	19.8	269.6	6.83	6.09
71	313.7	581.4	19.4	313.7	6.30	6.80
78	354.0	542.3	19.0	354.0	5.75	7.50
85	389.4	472.4	18.5	389.4	5.07	8.36

Note: *The initial time taken for the egg to hatch was assumed to be 22 days.
** Average weight of a one day old chick was assumed to be 40 g.

We can derive enzymes and enzyme complexes by using equation (15) to determine $[E_o^{'}]$ and equation (14) to obtain $[ES^{'}]$. Then, using equation (13) we can yield $[E^{'}]$ as given in **Table S5** and **Fig. S2**. Consequently, we obtained very useful and absolute relationships (**Fig. S3**), in which

$$[E^{'}]=2[S]^{0.33} \qquad (36) \text{ and}$$

$$[E^{'}]=2[P]^{0.25} \qquad (37).$$

Table S5.

Derivation of $\left[E^{'}\right]$ generation from body entity in relation to $\left[S\right]$ from experimental data of a hen growth

$k_1 = \frac{d[S]}{dt}$ (g/day)	$k_2 = k_1 \frac{[S]}{P}$ (g/day)	$K_m^{'}$ (g)	$v_m^{'}$ (g/day)	$k_1^{'} = k_1 K_m^{'} - k_2$ (g/day)	$\left[E_o^{'}\right] = \frac{v_m^{'}}{k_2}$ (g)	$\left[ES^{'}\right] = \frac{v^{'}}{k_2}$ (g)	$\left[E^{'}\right]$ (g)
0.7	0.3	15.9	2.9	11.2	10.1	5.0	5.0
3.7	1.1	172.9	9.2	638.9	8.6	1.7	6.9
5.5	1.3	312.8	13.7	1,704.9	10.8	2.2	8.6
6.3	1.3	414.1	16.2	2,588.2	12.9	3.0	10.0
7.1	1.3	534.0	18.7	3,776.2	14.8	3.6	11.2
6.9	1.1	568.2	19.3	3,936.4	16.8	4.7	12.1
6.8	1.1	604.7	19.8	4,129.3	18.7	5.8	12.9
6.3	0.9	581.4	19.4	3,663.7	20.9	7.3	13.6
5.7	0.8	542.3	19.0	3,115.0	23.4	9.2	14.1
5.1	0.7	472.4	18.5	2,394.4	26.6	12.0	14.6

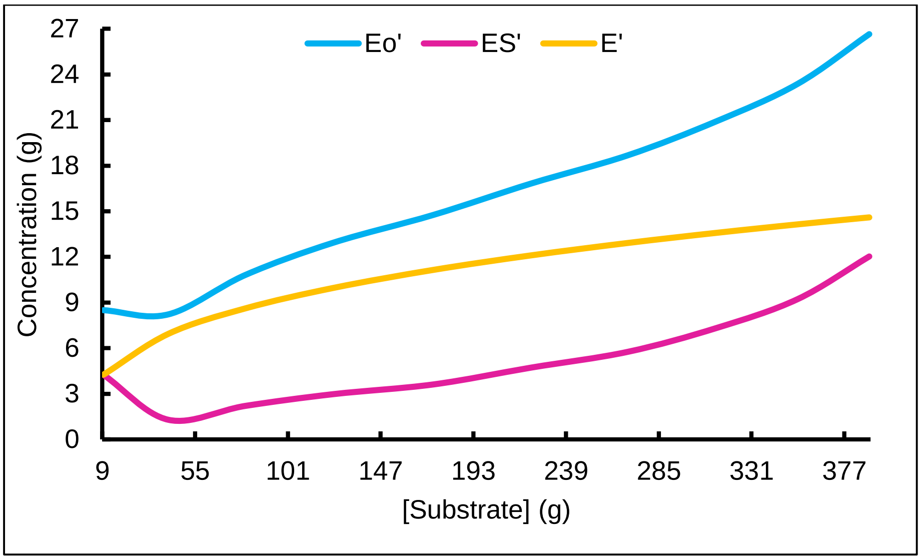

Figure S2.

The generation of $\left[E^{'}\right]$in relation to substrate $\left[S\right]$ of body entity in a hen growth

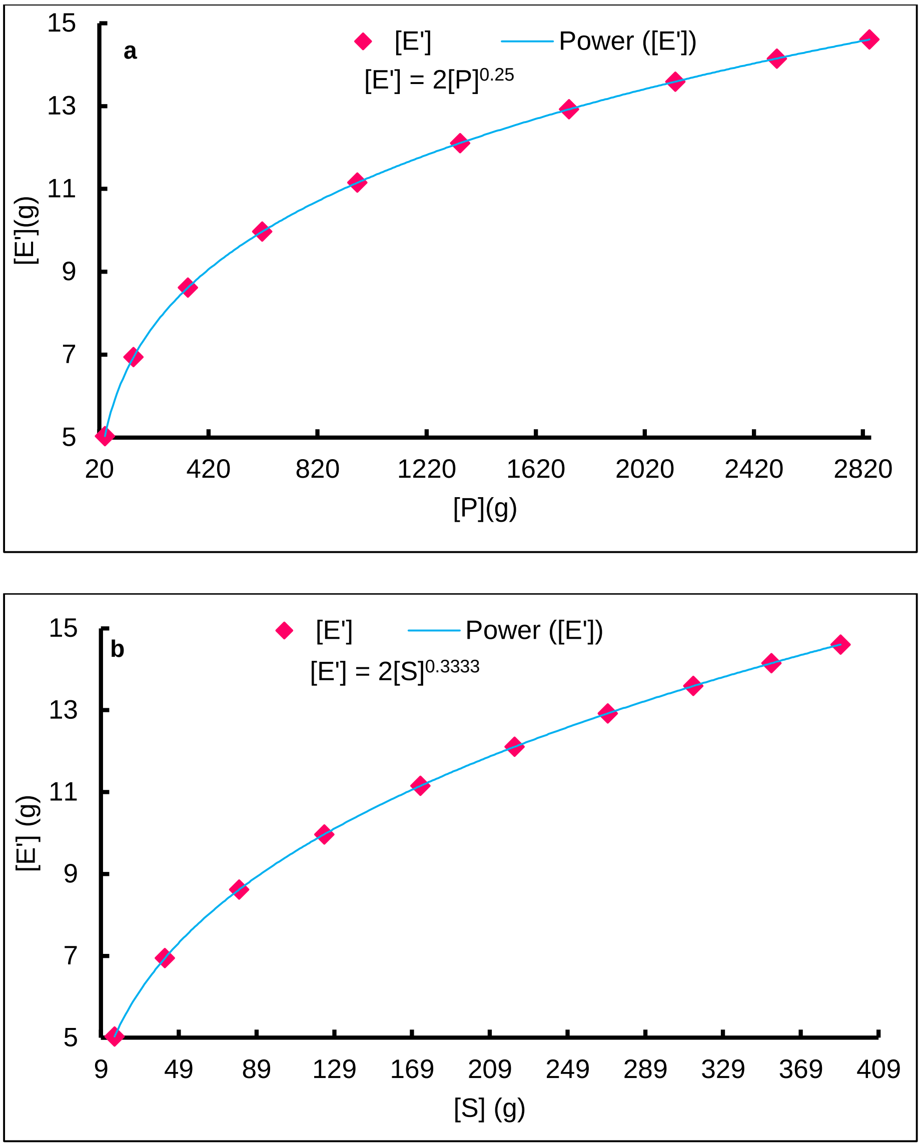

Figure S3.
Relationship between $[P]$ and $[E^{'}]$, $[S]$ and $[E^{'}]$ of a hen growth (a) Relationship between $[P]$ and $[E^{'}]$ of a hen growth (b) Relationship between $[S]$ and $[E^{'}]$

Analysis of Path Y - External Food and Nutrients Supply

Supplementary method 4
Derivation of $v_m^{''}$ and $K_m^{''}$ at time *t*

$v_m^{''}$ and $K_m^{''}$ at time t were calculated (**Table S6**) from the following equations which were derived by rearranging equation (41) .

$$\frac{dv}{dt} = \frac{0.5\sqrt{v_m^{''} K_m^{''}}}{t^{1.5}} \qquad (41)$$

$$v_m^{''} = \left(\frac{t}{0.5} \frac{dv}{dt} \right) + v \qquad (S8)$$

$$K_m^{''} = \frac{t\left(v_m^{''} - v\right)^2}{v_m^{''}} \qquad (S9)$$

Table S6.
Derivation of $K_m^{''}$ and $v_m^{''}$ for simulating reaction rate (*v*) of a hen growth

Time (days)	$[S]$ *(g)*	v (g/day)	$v_m^{''}$ (g/day)	$K_m^{''}$ (g)	$v = v_m^{''} - \sqrt{\frac{v_m^{''} K_m^{''}}{t}}$ (g/day)	$v^{''} = \frac{v_m^{''}[S]}{K_m^{''} + [S]}$ (g/day)
22	15.9	0.72	2.2	21.2	0.72	0.93
29	41.8	1.62	9.0	176.2	1.62	1.73
36	80.0	2.68	13.6	315.4	2.68	2.75
43	123.8	3.70	16.2	415.2	3.70	3.72
50	173.3	4.69	18.8	531.3	4.69	4.63
57	221.8	5.54	19.4	564.5	5.54	5.47
64	269.6	6.28	19.9	598.9	6.28	6.19
71	313.7	6.91	19.5	578.3	6.91	6.86
78	354.0	7.42	18.9	544.6	7.42	7.45
85	389.4	7.84	18.0	486.1	7.84	8.00

We sought to verify by multiplication of derivatives to determine exact values of $\frac{d[S]}{dt}$ as given in **Table S7.**

Table S7.

Verification of $\frac{d[S]}{dt}$ with the multiplication of derivatives of a hen growth data

Time (t) (days) $t=\frac{d[S]}{dv}$	$[S]$ (g)	$\frac{d[S]}{dt}=\frac{0.5K'_m v'_m}{\sqrt{K'_m v'_m t}}$	$\frac{dv}{dt}=\frac{0.5\sqrt{v''_m K''_m}}{t^{1.5}}$	$\frac{d[S]}{dt}=\frac{d[S]}{dv}\cdot\frac{dv}{dt}$
22	15.9	0.72	0.03	0.72
29	41.8	3.70	0.13	3.70
36	80.0	5.46	0.15	5.46
43	123.8	6.25	0.15	6.25
50	173.3	7.07	0.14	7.07
57	221.8	6.93	0.12	6.93
64	269.6	6.83	0.11	6.83
71	313.7	6.30	0.09	6.30
78	354.0	5.75	0.07	5.75
85	389.4	5.07	0.06	5.07

As before, we used equation (15), equation (14), and equation (13) to obtain the amounts of initial enzymes $[E''_o]$, enzyme complexes $[ES'']$, and $[E'']$ as given in **Table S8** and **Fig. S4**.

Table S8.
Derivation of $\left[E''\right]$ generation from the substrate governing the reaction of a hen growth

$k_1 = \frac{d[S]}{dt}$ (g/day)	$k_2 = k_1 \frac{[S]}{P}$ (g/day)	v''_m (g/day)	K''_m (g)	$k''_1 = k_1 K''_m - k_2$ (g/day)	$\left[E''_o\right]$ (g)	$\left[ES''\right]$ (g)	$\left[E''\right]$ (g)
0.7	0.3	2.2	21.2	15.0	7.5	3.2	4.3
3.7	1.1	9.0	176.2	651.3	8.5	1.6	6.8
5.5	1.3	13.6	315.4	1,719.2	10.7	2.2	8.6
6.3	1.3	16.2	415.2	2,595.2	12.9	3.0	9.9
7.1	1.3	18.8	531.3	3,757.1	14.8	3.7	11.2
6.9	1.1	19.4	564.5	3,910.8	16.9	4.8	12.2
6.8	1.1	19.9	598.9	4,089.5	18.9	5.9	13.0
6.3	0.9	19.5	578.3	3,644.6	21.0	7.4	13.6
5.7	0.8	18.9	544.6	3,128.0	23.3	9.2	14.1

Note:

$$\left[E''_o\right] = \frac{v''_m}{k_2}$$

$$\left[ES''\right] = \frac{v''}{k_2}$$

$$\left[E''\right] = \left[E''_o\right] - \left[ES''\right]$$

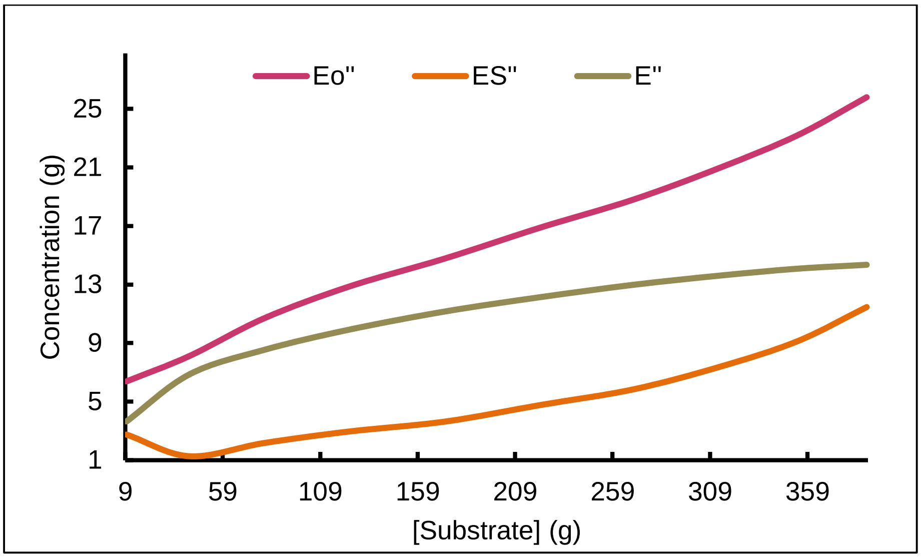

Figure S4.
The generation of enzymes $\left[E''\right]$ from the substrate governing the reaction of a hen growth

Combined Function and Inhibitions

We can state that the two quadratic functions (equation (33) and equation (40)) are responsible for the generations of enzymes. . In other words, the total of enzymes ($\left[E^{'''}\right]=\left[E^{'}\right]+\left[E^{''}\right]$) and enzyme complexes ($\left[ES^{'''}\right]=\left[ES^{'}\right]+\left[ES^{''}\right]$), thus $\left[E_o^{'''}\right]=\left[E_o^{'}\right]+\left[E_o^{''}\right]$ (**Table S9**) are the total generations giving another set of $v_m^{'''}$ and $K_m^{'''}$, since $v^{'''}=v^{'}+v^{''}$.

Table S9.

Calculation of $\left[E_o^{'''}\right]$, $\left[ES^{'''}\right]$, and $\left[E^{'''}\right]$ of growth of a hen

$\left[E_o^{'}\right]$ (g)	$\left[E_o^{''}\right]$ (g)	$\left[E_o^{'''}\right]$ (g)	$\left[E_o^{'''}\right]^*$ (g)	$\left[ES^{'}\right]$ (g)	$\left[ES^{''}\right]$ (g)	$\left[ES^{'''}\right]=\frac{v^{''}+v^{'}}{k_2}$ (g)	$\left[ES^{'''}\right]^*=\left[ES^{'}\right]+\left[ES^{''}\right]$ (g)	$\left[E^{'''}\right]=\left[E^{'}\right]+\left[E^{''}\right]$ (g)
10.1	7.5	17.6	17.6	5.0	3.2	8.3	8.3	9.3
8.6	8.5	17.1	17.1	1.7	1.6	3.3	3.3	13.8
10.8	10.7	21.6	21.6	2.2	2.2	4.4	4.4	17.2
12.9	12.9	25.9	25.9	3.0	3.0	5.9	5.9	19.9
14.8	14.8	29.6	29.6	3.6	3.7	7.3	7.3	22.3
16.8	16.9	33.8	33.8	4.7	4.8	9.5	9.5	24.3
18.7	18.9	37.5	37.5	5.8	5.9	11.6	11.6	25.9
20.9	21.0	42.0	42.0	7.3	7.4	14.7	14.7	27.2
23.4	23.3	46.7	46.7	9.2	9.2	18.4	18.4	28.3
26.6	25.9	52.5	52.5	12.0	11.5	23.6	23.6	29.0

Note: $\left[E_o^{'}\right]=\frac{v_m^{'}}{k_2}$

$\left[E_o^{''}\right]=\frac{v_m^{''}}{k_2}$

$\left[E_o^{'''}\right]=\left[E_o^{'}\right]+\left[E_o^{''}\right]$

*Verification

$\left[E_o^{'''}\right]^*=\left[ES^{'''}\right]+\left[E^{'''}\right]$

$\left[ES^{'}\right]=\frac{v^{'}}{k_2}$

$\left[ES^{''}\right]=\frac{v^{''}}{k_2}$

We can presume existence of proportionate values of *v* from $v^{'}$ and $v^{''}$ persisting in the production of enzymes $[E]$ and $[ES]$ complexes (**Table S10 and Table S11**). Such that;

$$v=\alpha v+\beta v \qquad (42)$$

Where,

$$\alpha=\frac{v^{'}}{(v^{'}+v^{''})} \qquad (43)$$

$$\beta=\frac{v^{''}}{(v^{'}+v^{''})} \qquad (44)$$

Calculation of proportionate values enzymes and enzyme-substrate concentrations

Proportionate values of ES_α , E_α , and $E_{o\alpha}$ and ES_β , E_β , and $E_{o\beta}$ can be found based on equation (42), equation (43) and equation (44) as given in **Table S10**.

Supplementary method 5
Calculation of proportionate values of enzymes and enzyme-substrate concentrations
In applying equation (14), the proportionate ES complex can be found, where;

$$[ES_\alpha] = \frac{\alpha v}{k_2} \quad \text{(S10) and}$$

$$[E_\alpha] = [E'] \times \frac{[ES_\alpha]}{[ES']} \quad \text{(S11)}$$

Thus giving

$$[E_{o\alpha}] = [ES_\alpha] + [E_\alpha] \quad \text{(S12).}$$

Similarly

$$[ES_\beta] = \frac{\beta v}{k_2} \quad \text{(S13),}$$

$$[E_\beta] = [E''] \times \frac{[ES_\beta]}{[ES'']} \quad \text{(S14) and}$$

$$[E_{o\beta}] = [ES_\beta] + [E_\beta] \quad \text{(S15).}$$

$$[ES] = [ES_\alpha] + [ES_\beta] \quad \text{(S16)}$$

$$[E] = [E_\alpha] + [E_\beta] \quad \text{(S17)}$$

$$[E_o] = [E_{o\alpha}] + [E_{o\beta}] \quad \text{(S18)}$$

Supplementary method 6
Calculation of inhibitor concentration $[I]$
Under un-competitive inhibition conditions,

$$\frac{[I]}{k_i} = \frac{v_m}{v} - 1 - \frac{K_m}{[S]} \quad \text{(S19)}$$

Where, $[I]$= inhibitor concentration and

$$k_i = \frac{K_m''''}{K_m} = 1 \quad \text{(S20)}$$

Table S10.

Calculation of $[ES_\alpha]$, $[E_\alpha]$, $[ES_\beta]$, and $[E_\beta]$ of a hen growth

v' (g/day)	v'' (g/day)	$[ES_\alpha] = \frac{\alpha v}{k_2}$ (g)	$[E_\alpha] = [E'] \times \frac{[ES_\alpha]}{[ES']}$ (g)	$[ES_\beta] = \frac{\beta v}{k_2}$ (g)	$[E_\beta] = [E''] \times \frac{[ES_\beta]}{[ES'']}$ (g)
1.45	0.93	1.53	1.53	0.98	1.31
1.79	1.73	0.77	3.19	0.74	3.14
2.79	2.75	1.07	4.16	1.05	4.14
3.74	3.72	1.48	4.94	1.47	4.93
4.59	4.63	1.84	5.67	1.85	5.69
5.41	5.47	2.40	6.16	2.43	6.19
6.09	6.19	2.95	6.61	3.00	6.65
6.80	6.86	3.71	6.87	3.74	6.89
7.50	7.45	4.58	7.02	4.55	7.00
8.36	8.00	5.77	7.00	5.52	6.89

Note: $v' = \frac{v'_m [S]}{K'_m + [S]}$

$v'' = \frac{v''_m [S]}{K''_m + [S]}$

Table S11.

Calculation of $v_{m(\alpha+\beta)}$, $K_{m(\alpha+\beta)}$, and v of a hen growth

$[ES]$ (g)	$[E]$ (g)	$[E_o]$ (g)	$v_{m(\alpha+\beta)} = [E_o].k_2$ (g/day)	$K_{m(\alpha+\beta)} = \frac{[E][S]}{[ES]}$ (g)	v (g/day)
2.51	2.84	5.36	1.54	17.98	0.72
1.52	6.33	7.84	8.36	174.53	1.62
2.11	8.30	10.42	13.19	314.06	2.68
2.94	9.86	12.81	16.07	414.61	3.70
3.69	11.35	15.05	19.09	532.69	4.69
4.84	12.35	17.18	19.67	566.34	5.54
5.94	13.26	19.21	20.31	601.75	6.28
7.44	13.76	21.20	19.67	579.84	6.91
9.14	14.03	23.16	18.81	543.44	7.42
11.29	13.89	25.18	17.48	479.11	7.84

Note: $[ES] = [ES_\alpha] + [ES_\beta]$

$[E] = [E_\alpha] + [E_\beta]$

$[E_o] = [E_{o\alpha}] + [E_{o\beta}]$

$$v = \frac{v_{m(\alpha+\beta)}[S]}{K_{m(\alpha+\beta)} + [S]}$$

Lineweaver-Burke Plot (LBP) and Eadie-Hofstee plot (EHP)

The Lineweaver-Burke plot (LBP) of $\frac{1}{S} vs \frac{1}{v}$ and Eadie-Hofstee plot (EHP) of $v\, vs \frac{v}{S}$ gave accurate regressions, see **Fig. S5**. The average values of v_m and K_m are compared in **Table S12.**

Table S12.

Variations in average v_m and K_m values simulated from combined equation and Lineweaver-Burke and Eadie-Hofstee plots for the case of a hen's growth

Method	v_m	K_m	K_m/v_m
Lineweaver-Burk plot	11.09	230.73	20.81
Eadie-Hofstee plot	13.05	290.66	22.28
Combined Equation (average)	15.42	422.44	27.40

Note: The accuracy does differ between the methods of deriving v. **Table S13** gives the simulated values from the simulated method and predicted values. It is notable that the simulated values derived from v_m and K_m values give precisely experimental data.

.

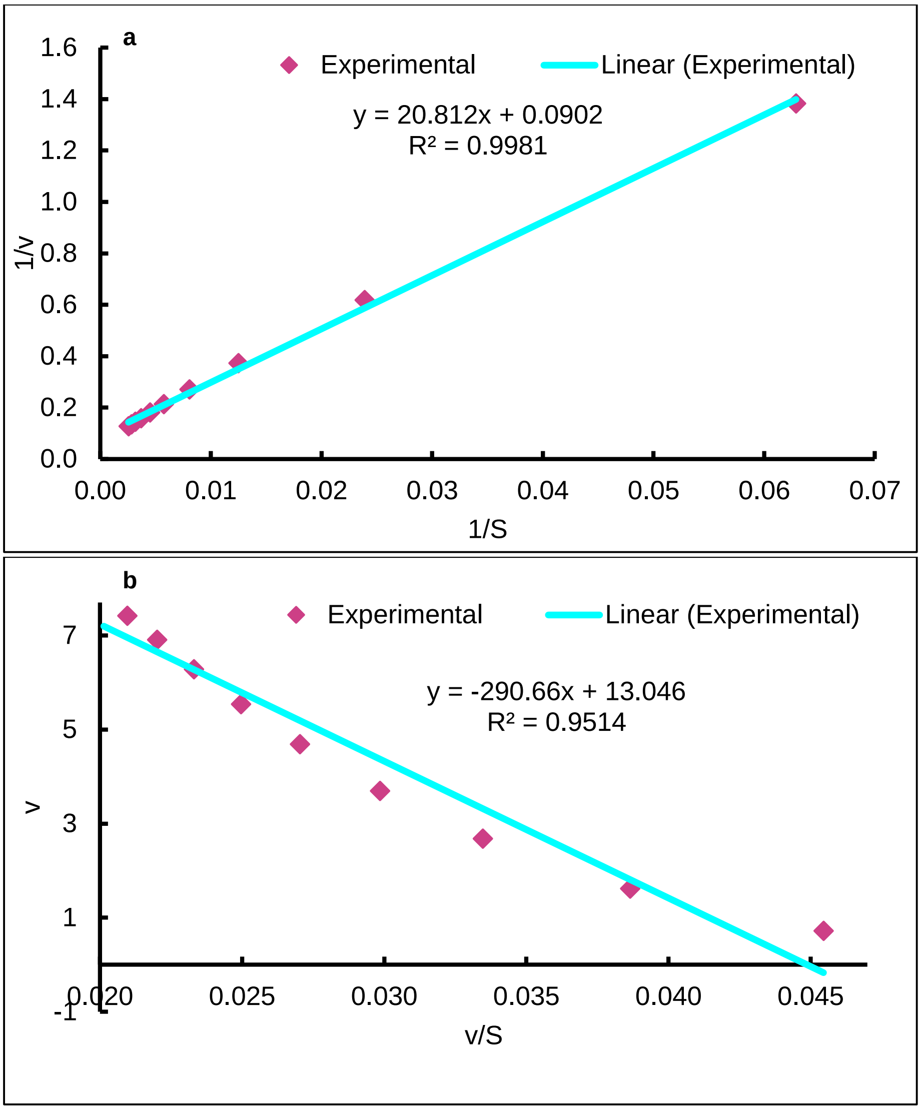

Figure S5.

Determination v_m and K_m of a hen growth data (**a**) Lineweaver-Burke plot (**b**) Eadie-Hofstee plot

Table S13.

Comparison of v deduced from Lineweaver-Burke plot (LBP), Eadie-Hofstee plot (EHP) and Combined Equation (CE)

Experimental v (g/day)	Simulated v (g/day)	Predicted v (g/day)		
		LBP	EHP	CE
0.72	0.72	0.71	0.68	0.56
1.62	1.62	1.70	1.64	1.39
2.68	2.68	2.85	2.82	2.46
3.70	3.70	3.87	3.90	3.5
4.69	4.69	4.76	4.87	4.49
5.54	5.54	5.43	5.65	5.31
6.28	6.28	5.97	6.28	6.01
6.91	6.91	6.39	6.77	6.57
7.42	7.42	6.71	7.16	7.03
7.84	7.84	6.96	7.47	7.40

Table S14.

Comparison of K_m/v_m deduced from Lineweaver-Burke plot (LBP), Eadie-Hofstee plot (EHP) and Combined Equation (CE) in different experiments

Experiment	K_m/v_m		
	LBP	EHP	CE
A hen's growth (*55*) : Weight changes in g from 22nd day to 85th day of age	20.81	22.28	27.40
Male-chick's growth (*55*): Weight changes in g from 22nd day to 85th day of age	20.94	22.61	28.86
Amaranthu soleraceus plant growth: plant height in cm as the growth parameter from 10th to 30th day after seedling emerged.	8.99	9.09	10.25
Cucumis sativus vine growth: vine length in cm as the growth parameter from 10th to 30th day after seedling emerged.	6.79	7.77	14.82
Solanum lycopersicum plant growth: plant height in cm as the growth parameter from 31st to 54th day after seedling emerged.	30.44	30.97	33.76
Blood glucose levels (mg/dL): blood glucose levels of a type 2 diabetes female patient were measured at 8 hour intervals for 72 hours.	5.55	5.67	6.56
Blood glucose levels (mg/dL): blood glucose levels of a female patient who was diagnosed with chronic kidney disease female patient were measured at 8 hour intervals for 224 hours.	5.90	6.20	9.55
Platelets count (E+03/dL) variations of a dengue patient after treatment of *Carica papaya* leaves extract were measured daily for 5 days (Ahmad et al., 2011).	0.71	0.72	0.91
Platelets count (E+03/dL) variations of dengue patient before treatment of C*arica papaya* leaves extract were measured daily for 5 days (Ahmad et al., 2011).	2.18	2.15	1.46
Variations of daily biochemical oxygen demand (BOD_1-BOD_6) (mg/L) of a dumpsite leachate sample were measured for 6 days.	0.76	0.79	1.16
A Solar Aeration Chimney composting system was filled with the organic fraction of the municipal solid waste. VS % (w/w) variations of the decomposing pile were analyzed daily for 25 days.	0.62	0.62	0.93
A Solar Aeration Chimney composting system was filled with a mixture of market waste, bio-char and Eppawela Rock Phosphate (ERP). VS % (w/w) variations of the mid stratum of the decomposing pile were analyzed daily for 60 days.	0.65	0.66	1.18
Total dissolved solids (mg/L) concentrations in an up-flow anaerobic sludge blanket reactor were measured daily for 30 days.	0.65	0.67	1.26
The volatile suspended solid concentrations (mg/L) of the elute of a hydrolysis phase reactor were measured for 30 days.	0.57	0.58	0.66

Application of developed formulism to experimental data
Example 02: Evaluation of the effects of biochar application on *Amaranthus oleraceus* plant growth

The field experiment was conducted in the Coconut Cultivation Board in Madamulana, Sri Lanka. It belongs to Hambanthota district and dry zone low country. Charcoal was produced from coconut shells by using pit method. The particle size of the produced charcoal was reduced and these materials were sieved by using 5 mm size sieve. *Amaranthus oleraceus* was selected as the planting material. 0.7 m x 2.15 m size planting bed was prepared. The soil conditions of the planting bed were analyzed for pH, moisture content, ash, total dissolved solids (TDS), electrical conductivity (EC), total nitrogen, phosphorus, and potassium, before planting, 10 days and 20 days after planting. Each plant height was measured firstly 10 days of planting and thereafter ones in two days for 30 days. The average height of the plants at time *t* throughout the experimental period was used for this study. Cumulative height (cm) at time *t* was calculated and it was considered as *P* for the study.

Table S15.
The calculation to derive *v* from *Amaranthus oleraceus* plant growth (experimental data)

Time (days)	Plant height (cm)	Cumulative plant height (P) (cm)	dP/dt (cm/day)	$[S]=[P]^n$ n = 0.75 (cm)	$d[S]$ (cm)	$\frac{d[S]}{t}$ (cm/day)	$v = \sum \frac{d[S]}{t}$ (cm/day)
10	9.4	9.4	0.94	5.3	5.35	0.53	0.53
12	10.3	19.6	5.13	9.3	3.97	0.33	0.87
14	11.4	31.0	5.69	13.1	3.82	0.27	1.14
16	12.5	43.5	6.26	16.9	3.81	0.24	1.38
18	14.0	57.5	6.98	20.9	3.93	0.22	1.59
20	16.0	73.5	7.99	25.1	4.22	0.21	1.81
22	17.5	90.9	8.73	29.4	4.35	0.20	2.00
24	19.5	110.4	9.73	34.1	4.61	0.19	2.20
26	21.5	131.9	10.73	38.9	4.85	0.19	2.38
28	22.7	154.5	11.33	43.8	4.92	0.18	2.56
30	23.7	178.2	11.85	48.8	4.95	0.17	2.72

Note:
It was assumed that cumulative plant height is proportionate to plant weight.

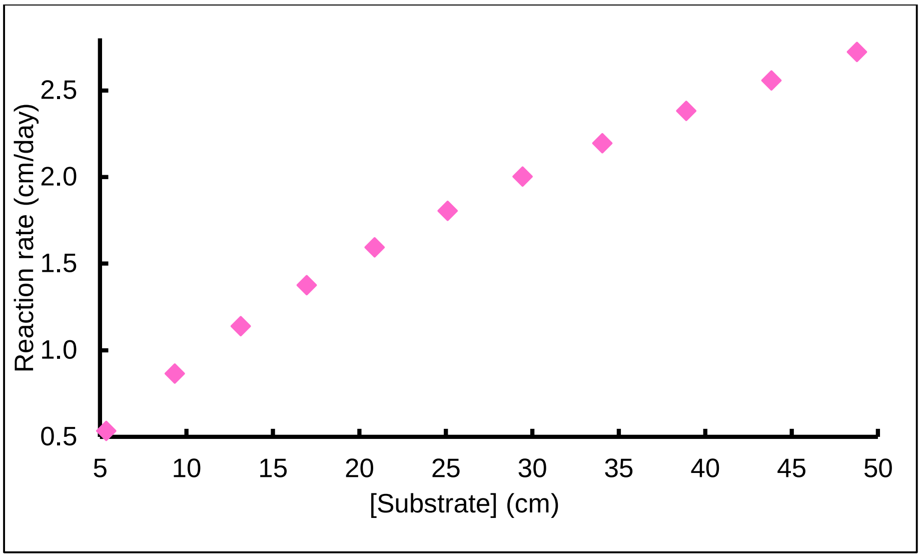

Figure S6.
Illustration of rate of reaction *v* as a function of substrate of *Amaranthus oleraceus* plant growth (cumulative height (cm) $\propto$ weight (g)) experiment

Table S16.

Derivation of $K_m^{'}$ and $v_m^{'}$ for simulating [S] of *Amaranthus oleraceus* plant growth

Time (days)	S (cm)	$K_m^{'}$ (cm)	$v_m^{'}$ (cm/day)	$[S]=\sqrt{v_m K_m t}-K_m$ (cm)	$\frac{d[S]}{dt}=\frac{0.5K_m^{'}v_m^{'}}{\sqrt{K_m^{'}v_m^{'}t}}$	$v^{'}=\frac{v_m^{'}[S]}{K_m^{'}+[S]}$ (cm/day)
10	5.35	5.35	2.14	5.35	0.53	1.07
12	9.32	38.34	4.94	9.32	1.99	0.97
14	13.14	40.32	5.06	13.14	1.91	1.24
16	16.94	43.95	5.27	16.94	1.90	1.47
18	20.88	49.91	5.58	20.88	1.97	1.64
20	25.09	59.25	6.00	25.09	2.11	1.79
22	29.45	66.31	6.29	29.45	2.18	1.93
24	34.06	76.60	6.66	34.06	2.31	2.05
26	38.91	87.30	7.02	38.91	2.43	2.16
28	43.83	93.82	7.21	43.83	2.46	2.30
30	48.78	99.76	7.37	48.78	2.48	2.42

Note: It was assumed that cumulative plant height is proportionate to plant weight.

Table S17.
Derivation of $[E']$ generation from body entity in relation to $[S]$ from experimental data of *Amaranthus oleraceus* plant growth

$k_1 = \frac{d[S]}{dt}$ (cm/day)	$k_2 = k_1 \frac{[S]}{P}$ (cm/day)	K'_m (cm)	v'_m (cm/day)	$k'_1 = k_1 K'_m - k_2$ (cm/day)	$[E'_o] = \frac{v'_m}{k_2}$ (cm)	$[ES'] = \frac{v'}{k_2}$ (cm)	$[E']$ (cm)
0.53	0.31	5.35	2.14	2.55	6.99	3.50	3.50
1.99	0.94	38.34	4.94	75.18	5.23	1.02	4.21
1.91	0.81	40.32	5.06	76.18	6.26	1.54	4.72
1.90	0.74	43.95	5.27	82.89	7.12	1.98	5.14
1.97	0.71	49.91	5.58	97.41	7.81	2.30	5.51
2.11	0.72	59.25	6.00	124.20	8.34	2.48	5.86
2.18	0.70	66.31	6.29	143.59	8.92	2.74	6.18
2.31	0.71	76.60	6.66	175.88	9.36	2.88	6.48
2.43	0.72	87.30	7.02	211.18	9.80	3.02	6.78
2.46	0.70	93.82	7.21	229.92	10.35	3.29	7.05
2.48	0.68	99.76	7.37	246.30	10.88	3.57	7.31

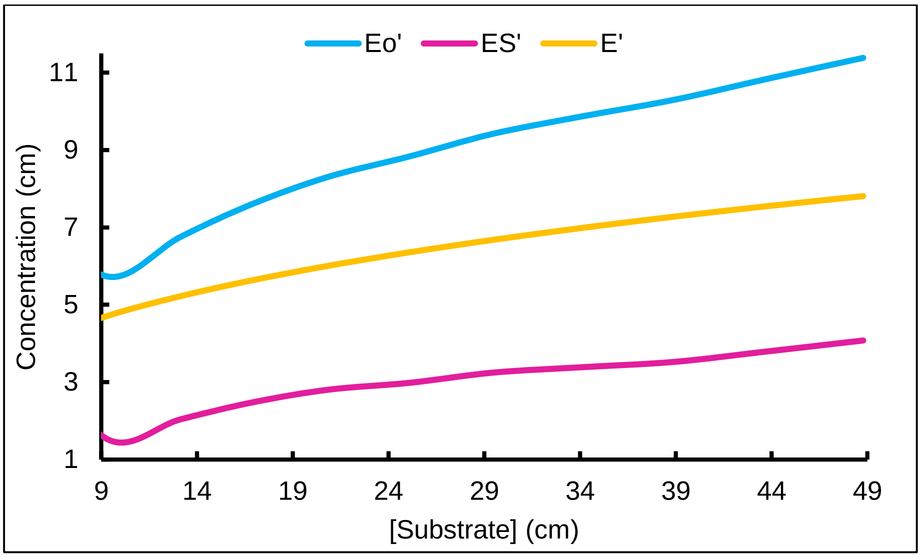

Figure S7.
The generation of $[E']$ in relation to substrate $[S]$ of body entity in *Amaranthus oleraceus* plant growth

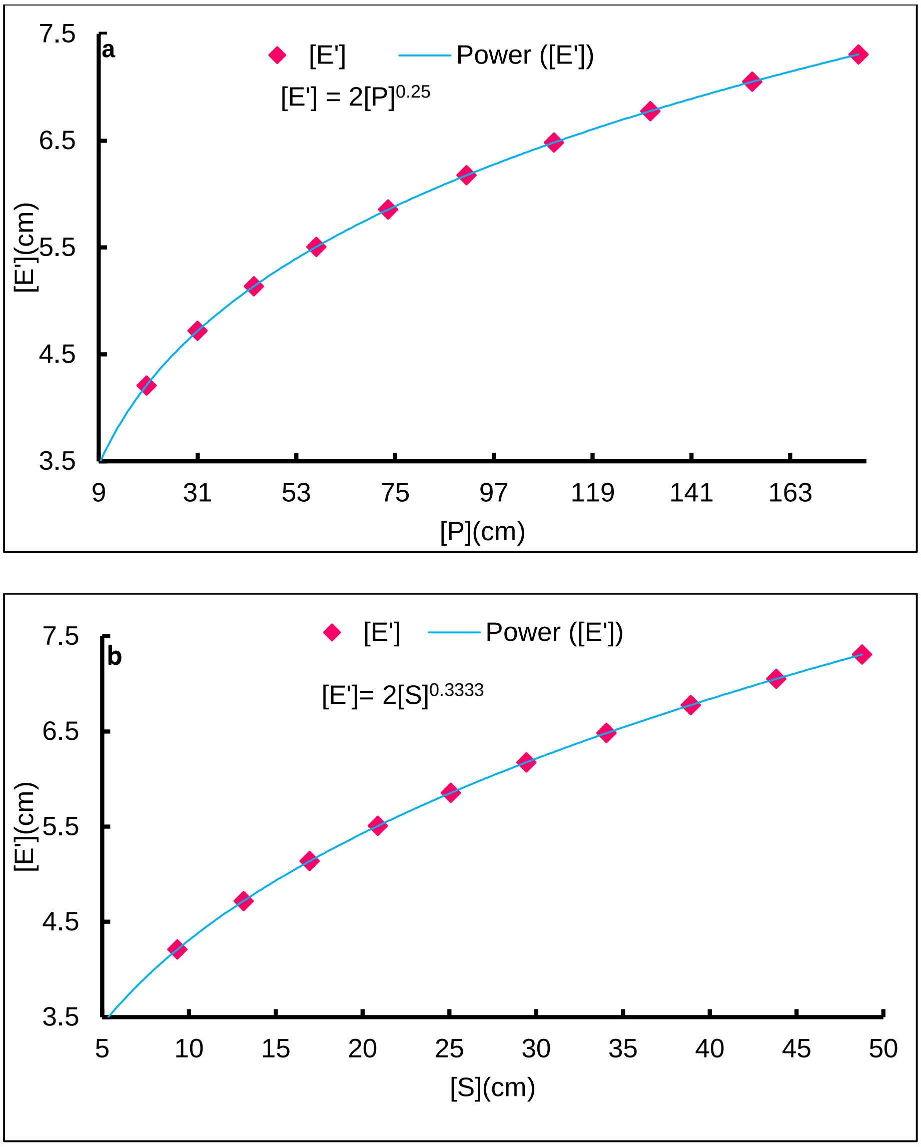

Figure S8.
Relationship between $[P]$ and $[E^{'}]$ *and* $[S]$ and $[E^{'}]$ *Aamaranthus oleraceus* plant growth (cumulative height (cm) α weight (g)) (a.) $[P]$ and $[E^{'}]$ (b.) $[S]$ and $[E^{'}]$

Table S18.

Derivation of $K_m^{"}$ and $v_m^{"}$ for simulating reaction rate (*v*) of the experimental data in *Amaranthus oleraceus* plant growth

Time (days)	$[S]$ (cm)	v (cm/day)	$v_m^{"}$ (cm/day)	$K_m^{"}$ (cm)	$v = v_m^{"} - \sqrt{\frac{v_m^{"} K_m^{"}}{t}}$ (cm/day)	$v^{"} = \frac{v_m^{"}[S]}{K_m^{"} + [S]}$ (cm/day)
10	5.3	0.53	1.60	7.13	0.53	0.69
12	9.3	0.87	4.84	39.13	0.87	0.93
14	13.1	1.14	4.96	41.18	1.14	1.20
16	16.9	1.38	5.18	44.72	1.38	1.42
18	20.9	1.59	5.53	50.36	1.59	1.62
20	25.1	1.81	6.02	59.05	1.81	1.80
22	29.4	2.00	6.36	65.57	2.00	1.97
24	34.1	2.20	6.81	74.96	2.20	2.13
26	38.9	2.38	7.24	84.66	2.38	2.28
28	43.8	2.56	7.47	90.54	2.56	2.44
30	48.8	2.72	7.67	95.84	2.72	2.59

Table S19.

Verification of $\frac{d[S]}{dt}$ with the multiplication of derivatives of *Amaranthus oleraceus* plant growth experiment

Time (t) (days) $t=\frac{d[S]}{dv}$	$[S]$ (cm)	$\frac{d[S]}{dt}=\frac{0.5K_m^{'}v_m^{'}}{\sqrt{K_m^{'}v_m^{'}t}}$	$\frac{dv}{dt}=\frac{0.5\sqrt{v_m^{''}K_m^{''}}}{t^{1.5}}$	$\frac{d[S]}{dt}=\frac{d[S]}{dv}.\frac{dv}{dt}$
10	5.3	0.53	0.05	0.53
12	9.3	1.99	0.17	1.99
14	13.1	1.91	0.14	1.91
16	16.9	1.90	0.12	1.90
18	20.9	1.97	0.11	1.97
20	25.1	2.11	0.11	2.11
22	29.4	2.18	0.10	2.18
24	34.1	2.31	0.10	2.31
26	38.9	2.43	0.09	2.43
28	43.8	2.46	0.09	2.46
30	48.8	2.48	0.08	2.48

Table S20.

Derivation of $\left[E''\right]$ generation from the substrate governing the reaction of *Amaranthus oleraceus* plant growth experiment

$k_1 = \frac{d[S]}{dt}$ (cm/day)	$k_2 = k_1 \frac{[S]}{P}$ (cm/day)	v''_m (cm/day)	K''_m (cm)	$k''_1 = k_1 K''_m - k_2$ (cm/day)	$\left[E''_o\right]$ (cm)	$\left[ES''\right]$ (cm)	$\left[E''\right]$ (cm)
0.53	0.31	1.60	7.13	3.51	5.25	2.25	3.00
1.99	0.94	4.84	39.13	76.75	5.13	0.99	4.14
1.91	0.81	4.96	41.18	77.82	6.13	1.48	4.64
1.90	0.74	5.18	44.72	84.36	6.99	1.92	5.07
1.97	0.71	5.53	50.36	98.30	7.74	2.27	5.47
2.11	0.72	6.02	59.05	123.79	8.36	2.49	5.87
2.18	0.70	6.36	65.57	141.99	9.02	2.79	6.22
2.31	0.71	6.81	74.96	172.10	9.57	2.99	6.58
2.43	0.72	7.24	84.66	204.77	10.10	3.18	6.92
2.46	0.70	7.47	90.54	221.85	10.72	3.50	7.22
2.48	0.68	7.67	95.84	236.58	11.33	3.82	7.51

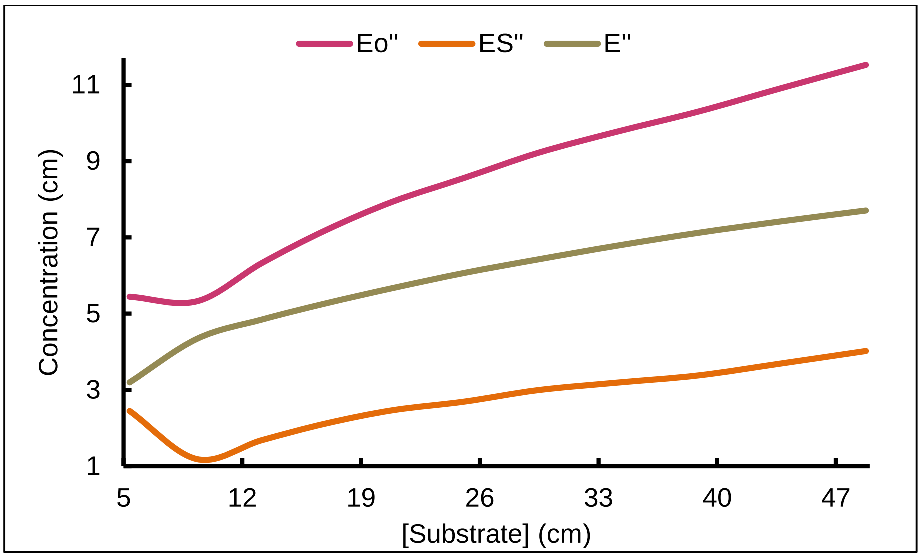

Figure S9.

The generation of enzymes $\left[E''\right]$ from the substrate governing the reaction in Amaranthus *oleraceus* plant growth

Table S21.

Calculation of $[E_o^{'''}]$, $[ES^{'''}]$, and $[E^{'''}]$ of *Amaranthus oleraceus* plants growth

$[E_o^{'}]$ (cm)	$[E_o^{''}]$ (cm)	$[E_o^{'''}]^*$ (cm)	$[E_o^{'''}]$ (cm)	$[ES^{'}]$ (cm)	$[ES^{''}]$ (cm)	$[ES^{'''}] = \frac{v^{''}+v^{'}}{k_2}$ (cm)	$[ES^{'''}] = [ES^{'}] + [ES^{''}]$ (cm)	$[E^{'''}] = [E^{'}] + [E^{''}]$ (cm)
6.99	5.25	12.24	12.24	3.50	2.25	5.75	5.75	6.50
5.23	5.13	10.36	10.36	1.02	0.99	2.01	2.01	8.35
6.26	6.13	12.38	12.38	1.54	1.48	3.02	3.02	9.36
7.12	6.99	14.11	14.11	1.98	1.92	3.90	3.90	10.21
7.81	7.74	15.55	15.55	2.30	2.27	4.57	4.57	10.98
8.34	8.36	16.70	16.70	2.48	2.49	4.97	4.97	11.72
8.92	9.02	17.94	17.94	2.74	2.79	5.54	5.54	12.40
9.36	9.57	18.93	18.93	2.88	2.99	5.87	5.87	13.06
9.80	10.10	19.90	19.90	3.02	3.18	6.20	6.20	13.70
10.35	10.72	21.07	21.07	3.29	3.50	6.79	6.79	14.28
10.88	11.33	22.21	22.21	3.57	3.82	7.39	7.39	14.81

Note: $[E_o^{'}] = \frac{v_m^{'}}{k_2}$

$$[E_o^{''}] = \frac{v_m^{''}}{k_2}$$

$$[E_o^{'''}] = [E_o^{'}] + [E_o^{''}]$$

*Verification

$$[E_o^{'''}]^* = [ES^{'''}] + [E^{'''}]$$

$$[ES^{'}] = \frac{v^{'}}{k_2}$$

$$[ES^{''}] = \frac{v^{''}}{k_2}$$

Table S22.

Calculation of $[ES_\alpha]$, $[E_\alpha]$, $[ES_\beta]$, and $[E_\beta]$ of *Amaranthus oleraceus* plant growth

v' (cm/day)	v'' (cm/day)	$[ES_\alpha]=\frac{\alpha v}{k_2}$ (cm)	$[E_\alpha]=[E']\times\frac{[ES_\alpha]}{[ES']}$ (cm)	$[ES_\beta]=\frac{\beta v}{k_2}$ (cm)	$[E_\beta]=[E'']\times\frac{[ES_\beta]}{[ES'']}$ (cm)
1.07	0.69	1.06	1.06	0.68	0.91
0.97	0.93	0.47	1.92	0.45	1.89
1.24	1.20	0.72	2.20	0.69	2.16
1.47	1.42	0.94	2.45	0.91	2.41
1.64	1.62	1.13	2.69	1.11	2.67
1.79	1.80	1.25	2.95	1.26	2.96
1.93	1.97	1.41	3.17	1.43	3.20
2.05	2.13	1.52	3.41	1.57	3.46
2.16	2.28	1.62	3.63	1.71	3.71
2.30	2.44	1.78	3.81	1.89	3.90
2.42	2.59	1.94	3.97	2.08	4.08

Note: $v' = \frac{v'_m[S]}{K'_m + [S]}$

$v'' = \frac{v''_m[S]}{K''_m + [S]}$

Table S23.

Calculation of $v_{m(\alpha+\beta)}$, $K_{m(\alpha+\beta)}$, and v of *Amaranthus oleraceus* plant growth

$[ES]$ (cm)	$[E]$ (cm)	$[E_o]$ (cm)	$v_{m(\alpha+\beta)} = [E_o].k_2$ (cm/day)	$K_{m(\alpha+\beta)} = \frac{[E][S]}{[ES]}$ (cm)	v (cm/day)
1.75	1.98	3.73	1.14	6.05	0.53
0.92	3.81	4.73	4.46	38.73	0.87
1.41	4.36	5.77	4.67	40.74	1.14
1.86	4.86	6.72	4.98	44.33	1.38
2.23	5.36	7.60	5.42	50.13	1.59
2.51	5.91	8.42	6.06	59.15	1.81
2.84	6.37	9.21	6.49	65.93	2.00
3.09	6.87	9.96	7.08	75.77	2.20
3.33	7.35	10.67	7.64	85.95	2.38
3.67	7.71	11.38	7.94	92.13	2.56
4.02	8.05	12.07	8.18	97.73	2.72

Note: $[ES] = [ES_\alpha] + [ES_\beta]$

$[E] = [E_\alpha] + [E_\beta]$

$[E_o] = [E_{o\alpha}] + [E_{o\beta}]$

$$v = \frac{v_{m(\alpha+\beta)}[S]}{K_{m(\alpha+\beta)} + [S]}$$

Generation of $\left[E^{'}\right]$ in relation to substrate $\left[S\right]$ of body entity and generation of enzymes $\left[E^{''}\right]$ from the substrate governing the reaction in different experiments

Generation of enzymes $\left[E^{'}\right]$in relation to substrate $\left[S\right]$ of body entity in different experiments is illustrated in **Fig. S10**. And generation of enzymes $\left[E^{''}\right]$ from the substrate governing the reaction in different experiments is illustrated in **Fig. S11**. Experimental procedures of data collection are given in data acquisition for the analysis section (**Supplementary method 1**).

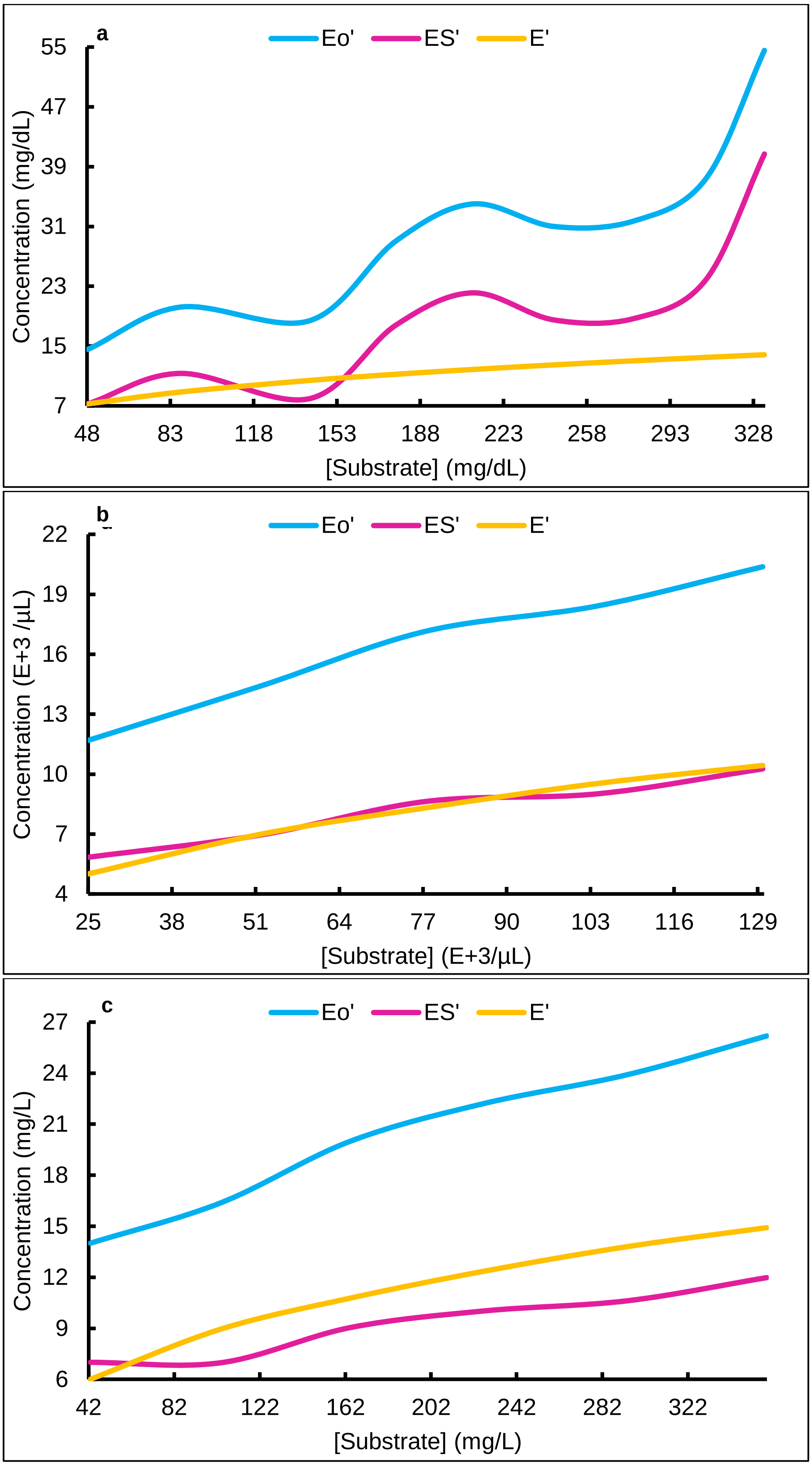

a
Eo'
ES'
E'
Concentration (mg/dL)
55
47
39
31
23
15
7
48
83
118
153
188
223
258
293
328
[Substrate] (mg/dL)
b
Eo'
ES'
E'
Concentration (E+3 /µL)
22
19
16
13
10
7
4
25
38
51
64
77
90
103
116
129
[Substrate] (E+3/µL)
c
Eo'
ES'
E'
Concentration (mg/L)
27
24
21
18
15
12
9
6
42
82
122
162
202
242
282
322
[Substrate] (mg/L)

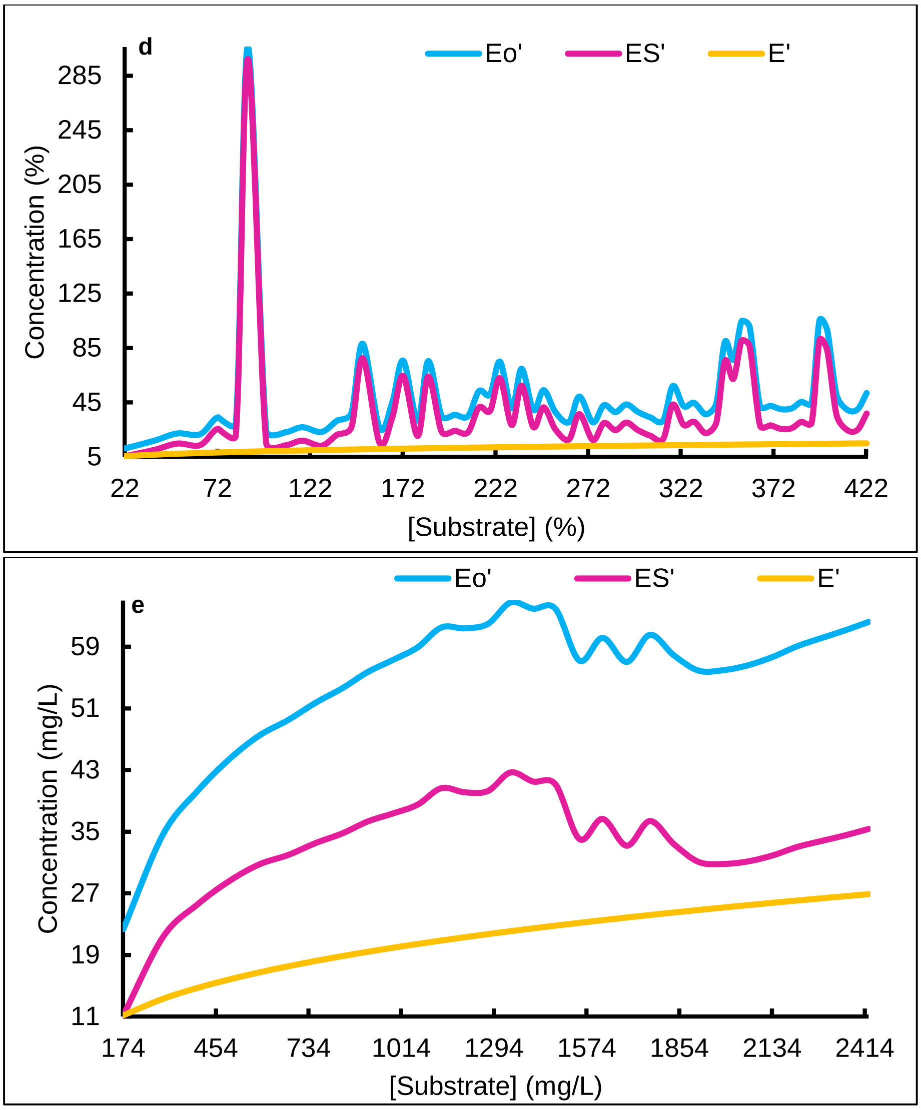

Figure S10.
The generation of $\left[E^{'}\right]$ in relation to substrate $\left[S\right]$ of body entity in different experiments (**a**) Levels of blood glucose variations of a type 2 diabetes female patient, (**b**) Platelets count variations of a dengue patient after treatment of *Carica papaya* leaves extract (Ahmad et al., 2011), (**c**) Variation of biochemical oxygen demand of dumpsite leachate, (**d**) VS % (w/w) as the substrate in a composting experiment, (**e**) Total dissolved solids (mg/L) concentration as the substrate in an up flow anaerobic sludge blanket reactor

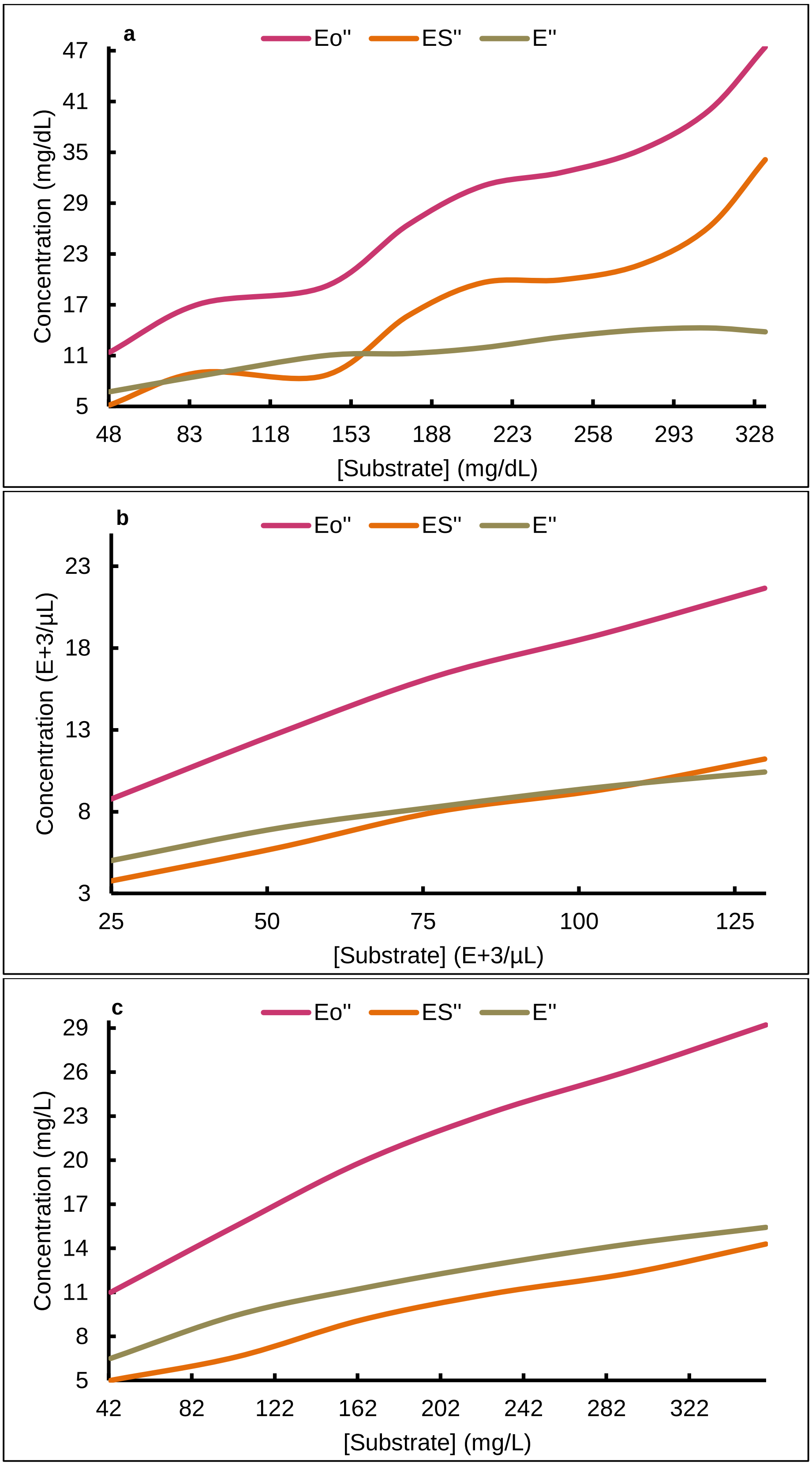
a
Eo''
ES''
E''
Concentration (mg/dL)
47
41
35
29
23
17
11
5
48
83
118
153
188
223
258
293
328
[Substrate] (mg/dL)
b
Eo''
ES''
E''
Concentration (E+3/µL)
23
18
13
8
3
25
50
75
100
125
[Substrate] (E+3/µL)
c
Eo''
ES''
E''
Concentration (mg/L)
29
26
23
20
17
14
11
8
5
42
82
122
162
202
242
282
322
[Substrate] (mg/L)

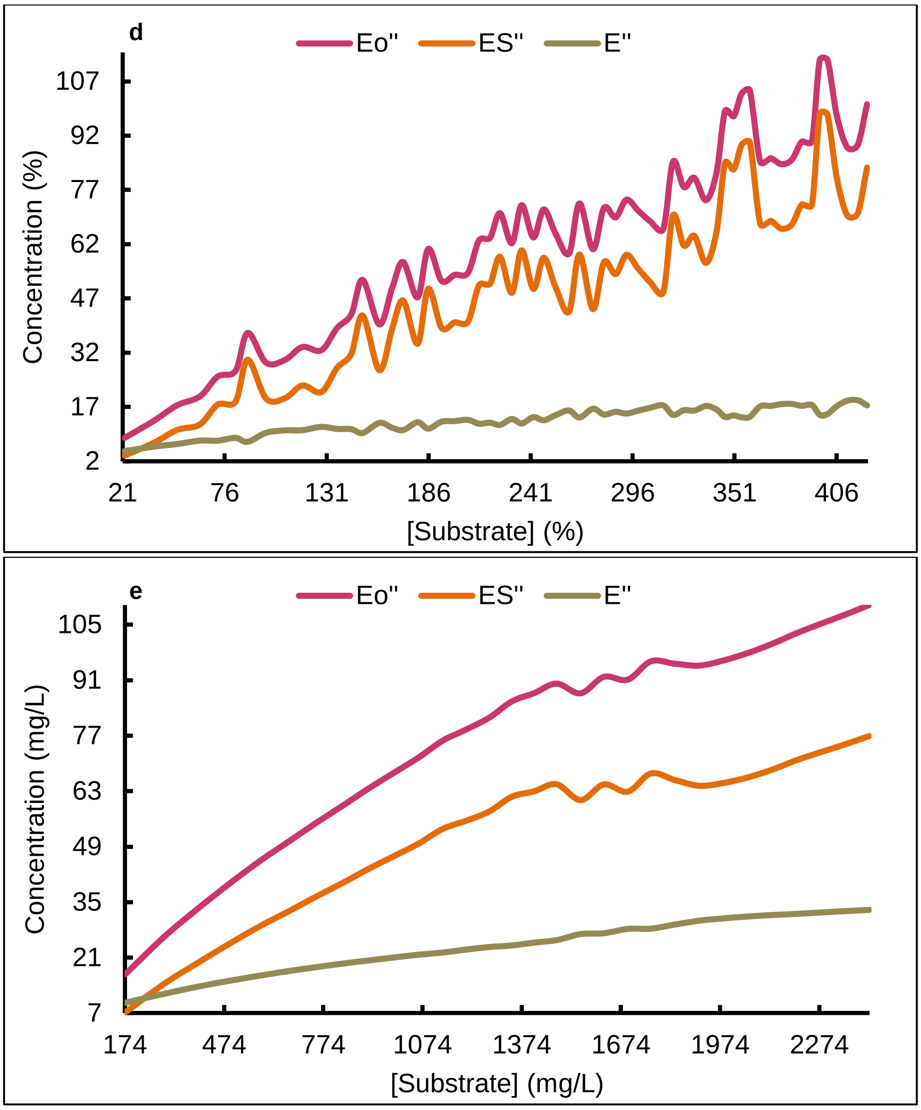

Figure S11.

The generation of enzymes $\left[E^{''}\right]$ from the substrate governing the reaction in different experiments (**a**) Levels of blood glucose variations of a type 2 diabetes female patient, (**b**) Platelets count variations of a dengue patient after treatment of *Carica papaya* leaves extract (Ahmad et al., 2011), (**c**) Variation of biochemical oxygen demand of dumpsite leachate, (**d**) VS % (w/w) as the substrate in a composting experiment, (**e**) Total dissolved solids (mg/L) concentration as the substrate in an up flow anaerobic sludge blanket reactor

Rates of change of variations of substrate concentrations, enzyme concentrations, enzyme substrate concentrations time of a type 2 diabetes patient (female) with time

In our analysis, we did not encounter negative values in $[S]$, $\frac{d[S]}{dt}$, $[E]$, k_1, and k_2, except in the case of diabetic patient of $-[E]$, but did surface negative values as outcomes in v_m, K_m, all forms of $[E_o]$, $[ES]$, $\frac{d[E]}{dt}$, $\frac{d[ES]}{dt}$ of a diabetic case (**Fig. S12**). Experimental procedures of data collection are given in data acquisition for the analysis section (Supplementary method 1).

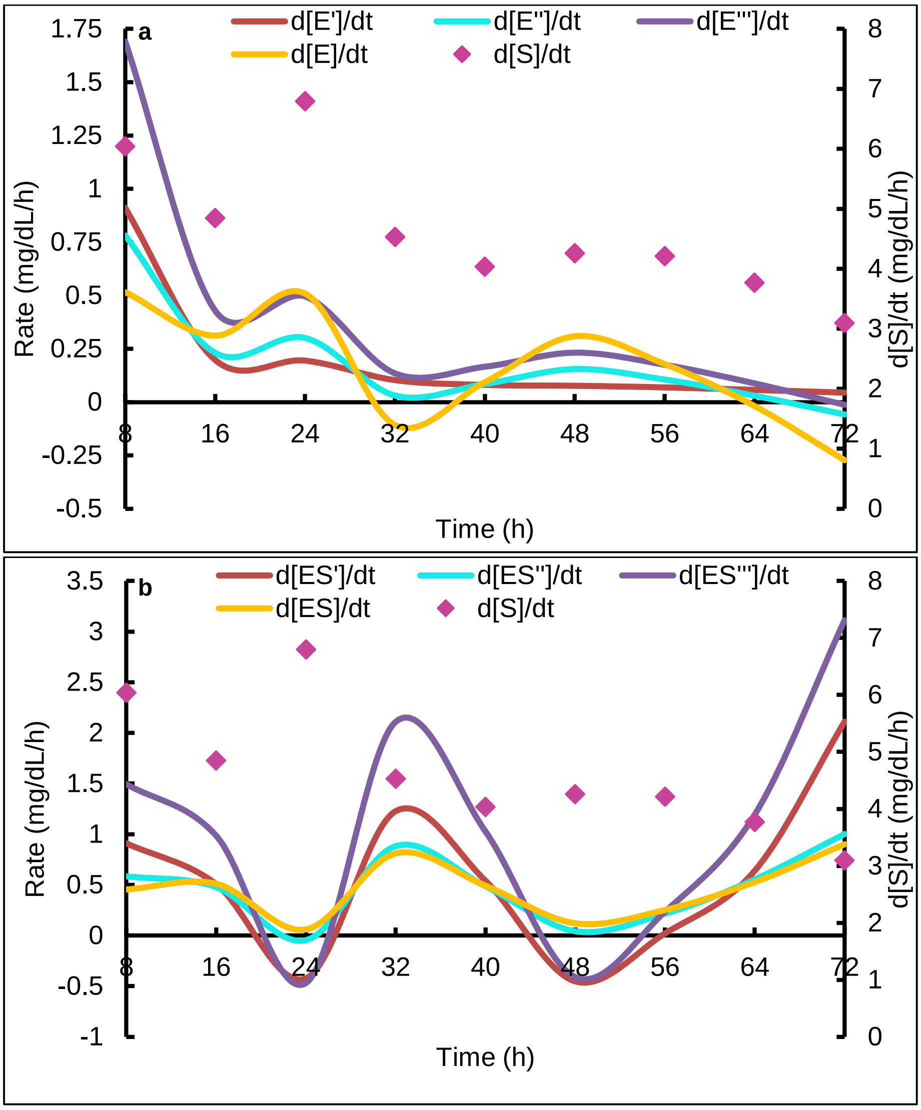

Figure S12.

Rates of change of variations of substrate concentrations $\frac{d[S]}{dt}$, enzyme concentrations ($\frac{d[E]}{dt}$), enzyme substrate concentrations($\frac{d[ES]}{dt}$) time of a type 2 diabetes patient (female) with time (**a**) $\frac{d[E]}{dt}$ (**b**) $\frac{d[ES]}{dt}$

Analysis of weight changes of a Caucasian, regaining weight in the dieting regime

A 57 year old male having an average weight of 99 kg body weight was recorded by himself for 31 days daily at the same time of the day. He had recorded the weight during a dieting program that he underwent. The written consent of the individual was obtained after explaining the objectives of the research. Cumulative weight (kg) at time *t* was calculated and it was considered as *P* for the study. The analysed results are illustrated in **Fig. S13**.

$-\frac{d\left[ES^{'}\right]}{dt}$ in a dieting regime can mean much less enzymes production corresponding to the loss in weight.

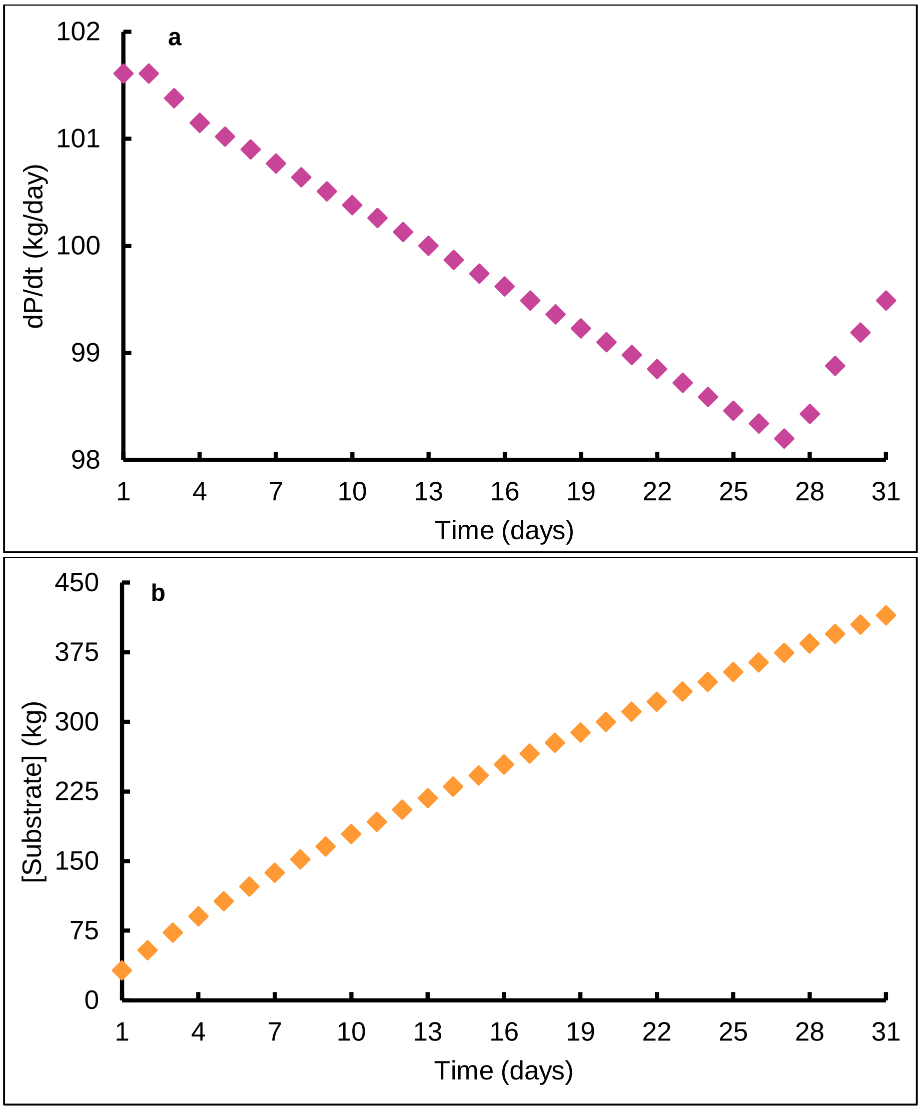

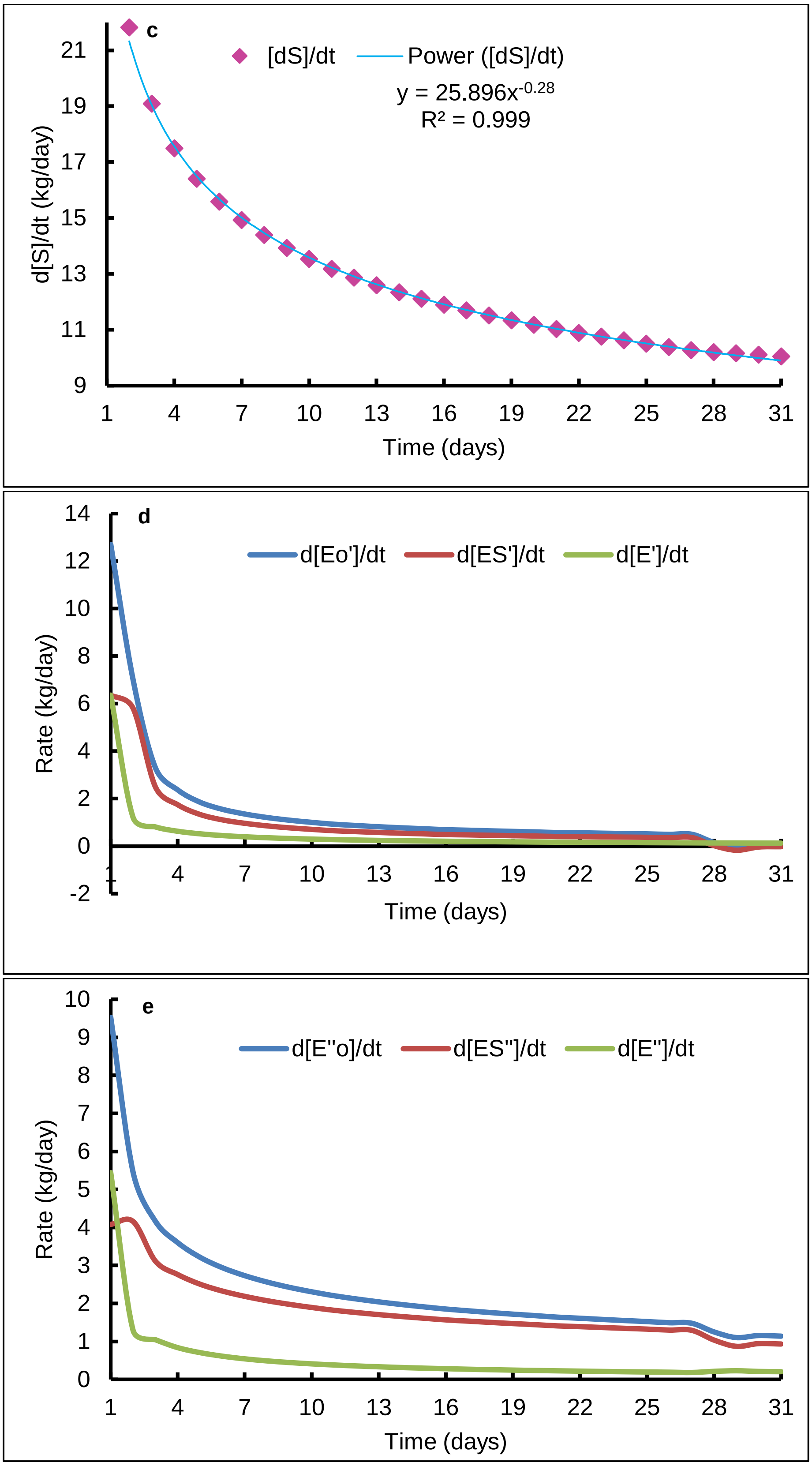
c
[dS]/dt
Power ([dS]/dt)
y = 25.896x-0.28
R² = 0.999
d[S]/dt (kg/day)
Time (days)
d
d[Eo']/dt
d[ES']/dt
d[E']/dt
Rate (kg/day)
Time (days)
e
d[E"o]/dt
d[ES"]/dt
d[E"]/dt
Rate (kg/day)
Time (days)

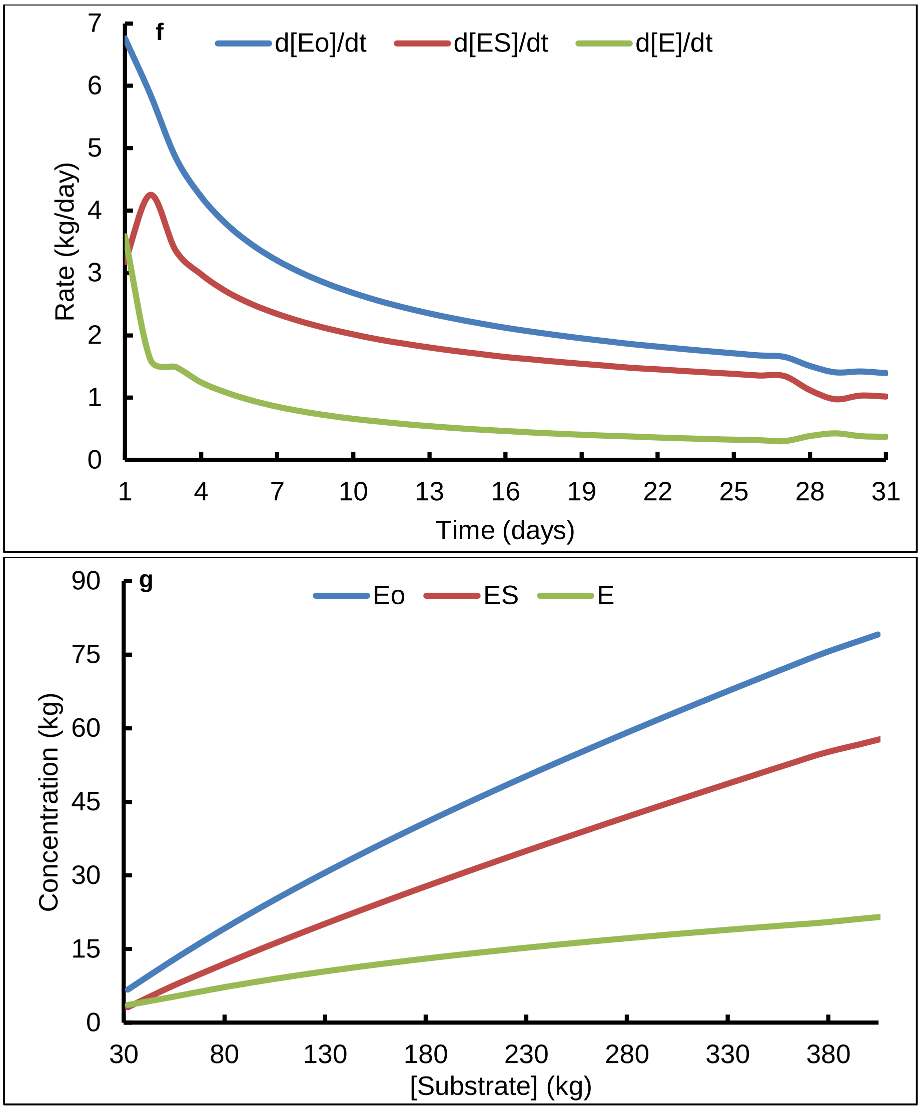

Figure S13.

Weight changes of a Caucasian, regaining weight in the dieting regime (**a**) $\frac{dP}{dt}$ variations with time (**b**) [Substrate] variations with time (**c**) $\frac{d[S]}{dt}$ variations with time (**d**) $\frac{d[E_o^{'}]}{dt}$, $\frac{d[ES^{'}]}{dt}$, and $\frac{d[E^{'}]}{dt}$ variations with time (**e**) $\frac{d[E_o^{''}]}{dt}$, $\frac{d[ES^{''}]}{dt}$, and $\frac{d[E^{''}]}{dt}$ variations with time (**f**) $\frac{d[E_o]}{dt}$, $\frac{d[ES]}{dt}$, and $\frac{d[E]}{dt}$ variations with time (**g**) $[E_o]$, $[ES]$, and $[E]$ variations with [Substrate]
